# *Pseudomonas* Lipopeptide-Mediated Biocontrol: Chemotaxonomy and Biological Activity

**DOI:** 10.3390/molecules27020372

**Published:** 2022-01-07

**Authors:** Feyisara Eyiwumi Oni, Qassim Esmaeel, Joseph Tobias Onyeka, Rasheed Adeleke, Cedric Jacquard, Christophe Clement, Harald Gross, Essaid Ait Barka, Monica Höfte

**Affiliations:** 1 Université de Reims Champagne Ardenne, Unité de Recherche RIBP EA4707 USC INRAE 1488, SFR Condorcet FR CNRS 3417, 51100 Reims, France; qassim.esmaeel@univ-reims.fr (Q.E.); cedric.jacquard@univ-reims.fr (C.J.); christophe.clement@univ-reims.fr (C.C.); ea.barka@univ-reims.fr (E.A.B.); 2 Department of Biological Sciences, Faculty of Science, Anchor University, Ayobo P.M.B 00001, Lagos State, Nigeria; 3 Unit for Environmental Sciences and Management, Faculty of Natural and Agricultural Sciences, North-West University, Potchefstroom 2520, South Africa; rasheed.adeleke@nwu.ac.za; 4 Plant Pathology Unit, National Root Crops Research Institute (NRCRI), Umudike 440001, Abia State, Nigeria; jonyeka@yahoo.com; 5 Department of Pharmaceutical Biology, Institute of Pharmaceutical Sciences, University of Tubingen, Auf der Morgenstelle 8, 72076 Tübingen, Germany; harald.gross@uni-tuebingen.de; 6 Laboratory of Phytopathology, Department of Plants and Crops, Faculty of Bioscience Engineering, Ghent University, Coupure Links 653, B-9000 Ghent, Belgium; monica.hofte@ugent.be

**Keywords:** secondary metabolites, lipopeptides, *Pseudomonas fluorescens*, antimicrobial, direct antagonism, induced systemic resistance, microbial competition, plant–pathogen interactions

## Abstract

*Pseudomonas* lipopeptides (Ps-LPs) play crucial roles in bacterial physiology, host–microbe interactions and plant disease control. Beneficial LP producers have mainly been isolated from the rhizosphere, phyllosphere and from bulk soils. Despite their wide geographic distribution and host range, emerging evidence suggests that LP-producing pseudomonads and their corresponding molecules display tight specificity and follow a phylogenetic distribution. About a decade ago, biocontrol LPs were mainly reported from the *P. fluorescens* group, but this has drastically advanced due to increased LP diversity research. On the one hand, the presence of a close-knit relationship between *Pseudomonas* taxonomy and the molecule produced may provide a startup toolbox for the delineation of unknown LPs into existing (or novel) LP groups. Furthermore, a taxonomy–molecule match may facilitate decisions regarding antimicrobial activity profiling and subsequent agricultural relevance of such LPs. In this review, we highlight and discuss the production of beneficial Ps-LPs by strains situated within unique taxonomic groups and the lineage-specificity and coevolution of this relationship. We also chronicle the antimicrobial activity demonstrated by these biomolecules in limited plant systems compared with multiple in vitro assays. Our review further stresses the need to systematically elucidate the roles of diverse Ps-LP groups in direct plant–pathogen interactions and in the enhancement of plant innate immunity.

## 1. Introduction

The *Pseudomonas* genus is ubiquitous and comprises species which are well known phytopathogens, such as *P. syringae*, or opportunistic human pathogens, such as *P. aeruginosa*, but also host members associated with water, soil and plant surfaces [1]. *Pseudomonas* spp. are well adapted to growing in the rhizosphere and are well suited for biocontrol and growth promotion [2]. Thus, the use of fluorescent *Pseudomonas* spp. as potential biopesticides has gained attention over the last decade. These bacteria are of particular interest because of their enormous metabolic versatility and wide adaptation across environmental gradients [3].

Based on phylogenomic and Multi Locus Sequence Analyses (MLSA), the *Pseudomonas* genus has been delineated into 453 species (https://lpsn.dsmz.de/genus/pseudomonas; accessed on 18 December 2021) which are distributed across three lineages (*P. fluorescens*, *P. aeruginosa* and *P. pertucinogena*), several groups (G) and subgroups (SG) [4,5,6,7,8]. Most biocontrol strains have been described within the *P. fluorescens* group comprising among others, the *P. fluorescens* SG, *P. koreensis* SG, *P. chlororaphis* SG, *P. jessenii* SG, *P. mandelii* SG and *P. corrugata* SG. Additionally, several biocontrol strains are positioned within the *P. putida* and *P. syringae* groups. These disease-suppressing pseudomonads were isolated from several sources ranging from the healthy plant rhizosphere [9,10,11], plant rhizosphere [12,13,14,15], phyllosphere [16,17], bulk soil [15] and suppressive soils [10,18]. The commonality among well-studied biocontrol strains is their capacity for secondary metabolite production including siderophores, lipopeptides (LPs), hydrogen cyanide, bacteriocins and certain antibiotics such as phenazines, 2,4-diacetylphloroglucinol (DAPG), pyrrolnitrin and pyoluteorin [3,19].

Examples of commercially available *Pseudomonas*-based bioprotectants include fungicides such as Cedomon and Cerall (*P. chlororaphis* MA342) both targeting seed-borne pathogens of cereals, Spot-Less (*P. aureofaciens* strain Tx-1) for management of fungal diseases on lawns and grasses, and Howler (*P. chlororaphis* AFS009) useful in the management of *Rhizoctonia*, *Pythium*, *Fusarium*, *Phytophthora*, *Colletotrichum* spp. in fruits, vegetables and ornamentals [19]. A detailed list of commercial bioprotectants based on *Pseudomonas* in Europe and USA, including their usage, and target crops/applications/pathogens have been enumerated in a recent review [19].

Lipopeptides are bacterial metabolites consisting of a peptide part attached to a fatty acid tail [1]. Most beneficial LPs are cyclized although linear LPs have also been described [20,21]. LPs have drawn remarkable interest because of their broad-spectrum antimicrobial and ecological functions. These multiple functions include biofilm formation and colonization of surfaces, quorum sensing, cell motility, soil remediation, anti-oomycete, antiviral, antifungal, antibacterial, herbicidal, insecticidal, antiprotozoal and anticancer properties [3,22,23,24,25,26,27].

In the past decade, the role of secondary metabolites, especially of LPs contributing to the biocontrol capability of *Pseudomonas* spp., has been increasingly studied [1,28]. This research wave was triggered by the increasing novelty of LPs that were being structurally and functionally characterized. On the one hand, several new LPs have been characterized within *Pseudomonas* groups [6,11,29] while the functions of some LPs have been recently characterized using insertion or deletion mutagenesis, crude LP extracts or purified LPs. Besides the review that highlighted the advances in *Pseudomonas* biocontrol [20], three recent reviews summarized so far the role of biosurfactants (mainly *Bacillus* LPs and rhamnolipids) in plant disease protection [30], described diverse elicitors of plant immunity produced by beneficial bacteria [31] and the use of *Pseudomonas* spp. as bacterial biocontrol agents to control plant disease [19]. The current review provides a summary of LP-producing biocontrol strains situated within specific *Pseudomonas* groups and highlights the taxonomy–molecule specificity of biocontrol Ps-LPs. Moreover, future areas of research and methodologies are proposed in order to accelerate our understanding of Ps-LP-based biocontrol and Ps-LP-pathogen interactions. Other aspects of LP-mediated plant–pathogen interactions are also discussed.

## 2. Methodology

In this review, bibliometric data were extracted from the SCOPUS database (https://www.scopus.com/; accessed on 20 February 2021) using the following specific keywords viscosin OR amphisin OR bananamide OR cocoyamide OR orfamide OR tolaasin OR syringomycin OR syringopeptin OR xantholysin OR putisolvin OR entolysin OR “cyclic lipopeptide” OR “cyclic lipopeptides” OR “CLPs” OR “lipopeptide” OR “lipopeptides” OR “LPs” AND *Pseudomonas* from which 118 documents were obtained. The bibliometric analysis was constructed using the VOSviewer processing software (v1.6.9., Leiden University, Leiden, The Netherlands).

A Comparative Genomic Blast Atlas was created using *Pseudomonas* genomes of 35 lipopeptide-producing strains, extracted from the National Centre of Biotechnology Information (NCBI) website. *P. fluorescens* Pf0-1 was chosen as the reference genome while representative LP-producing strains, selected from diverse *Pseudomonas* taxonomic groups/subgroups were uploaded as related genomes (Appendix A). As results, regions will be displayed where there is a similarity between the reference genome and one of the related genomes. Multi-Locus Sequence Analysis (MLSA) phylogenetic analysis was also conducted using the MEGAX software.

The literature search was conducted by accessing several databases including Scopus, PubMed, Web of Science, SpringerLink, Google Scholar and ResearchGate. A total of 126 articles and three book chapters were used. Schematic illustrations were drawn using the Biorender software.

**Figure 1:**
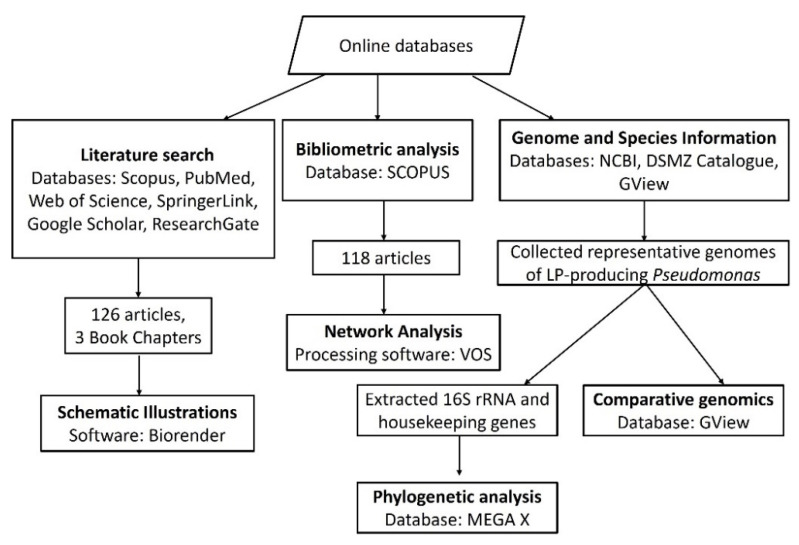
A detailed flow chart diagram describing the databases used and the study selection process.

For clarity, a series of detailed steps employed in writing this review is represented as a flowchart [32] (Figure 1).

## 3. Network Analysis Showing the Distribution of *Pseudomonas* LP-Related Articles

**Figure 2:**
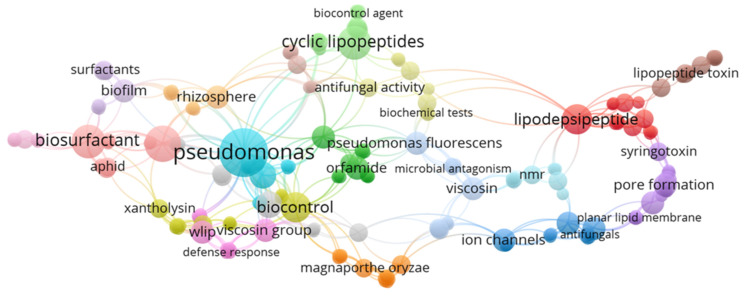
Bibliometric analysis for 118 papers published on cyclic lipopeptides of *Pseudomonas* according to the Scopus database using specific keywords such as viscosin OR amphisin OR bananamide OR cocoyamide OR orfamide OR tolaasin OR syringomycin OR syringopeptin OR xantholysin OR putisolvin OR entolysin AND *Pseudomonas* OR “cyclic lipopeptide” OR “cyclic lipopeptides” OR “CLPs” OR “lipopeptides” OR “lipopeptide” OR “LPs”.

The network analysis showed the distribution of articles related to cyclic lipopeptides, which helped to highlight the relationship between the keywords found and allowed a comprehensive perspective of the current research in this area (Figure 2). Clearly, these research areas will be enumerated in detail within this review.

## 4. Genome Comparison of Selected Lipopeptide-Producing *Pseudomonas* spp.

**Figure 3:**
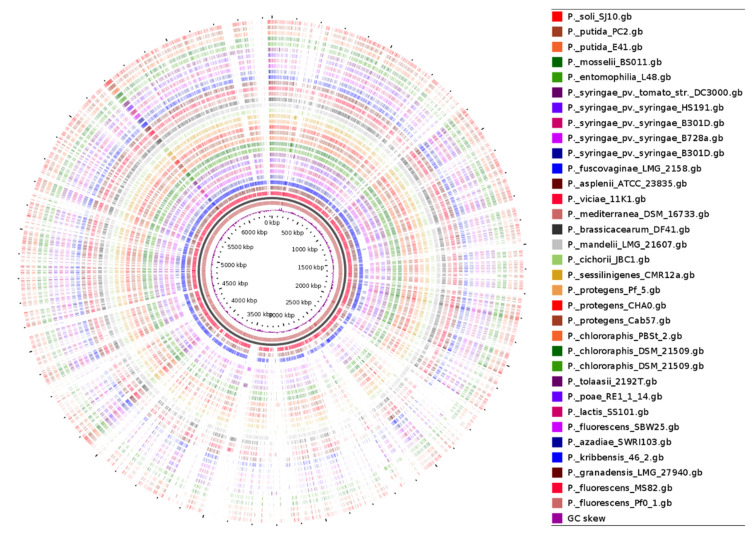
Comparative Genome Blast Atlas of 35 Lipopeptide-Producing *Pseudomonas* Strains. The BLAST Atlas analysis displays regions of the uploaded query files (34 genomes) where there are BLAST hits to the reference genome *P. fluorescens* Pf0-1). The GView Server was used [33].

A previous study provided the phylogenomic analysis of the *Pseudomonas* genus based on the genomes of the type strains of 163 described species and compared these type strain genomes to those of 1223 Pseudomonas genomes in public databases [7]. Results showed that 400 of those 1223 genomes were distinct from any other type strain suggesting that the *Pseudomonas* genomic diversity had been grossly underrepresented by the type strains. Furthermore, a detailed comparative genome analysis of ten strains within the *Pseudomonas fluorescens* group highlighted the enormous diverseness of this group and the capacity of the variable genome to adapt individual strains to their distinct lifestyles and functional capacities [3]. Here, using the *P. fluorescens* Pf0-1 as a reference genome, we compared the genome of 32 lipopeptide-producing *Pseudomonas* strains affiliated with the *P. koreensis*, *P. fluorescens*, *P. mandelii*, *P. corrugata*, *P. asplenii*, *P. chlororaphis*, *P. protegens*, subgroups including the *P. putida* and *P. syringae* groups. By comparing the protein coding sequences (CDS) of reference to query genomes, a Blast Atlas was generated which showed the close relatedness of other members of the *P. koreensis* group (*P. fluorescens* MS80, *P. granadensis* LMG 27,940 and *P. kribbensis* 46-2) to the reference genome *P. fluorescens* Pf0-1 (Figure 3). Clearly, these genomes are highly variable and distinct. Detailed comparative gene identities are presented in Table S1.

## 5. Chemical Diversity of Beneficial *Pseudomonas* LPs

**Figure 4:**
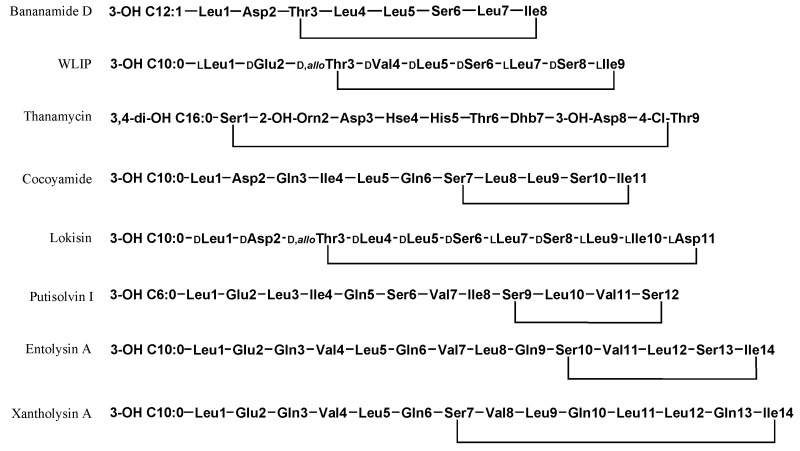
Chemical structures of selected biologically active *Pseudomonas* Cyclic Lipopeptides. Bananamide D (Bananamide Group); WLIP (Viscosin Group); Thanamycin (Syringomycin Group); Lokisin (Amphisin Group); Cocoyamide; Putisolvin I; Entolysin A and Xantholysin A. Whenever the absolute configuration of the lipopeptides was reported in the literature, it is indicated by standard stereodescriptors. In case of WLIP, the 3D-structure was secured by x-ray [69] and can be viewed as entry CCDC 919,229 at The Cambridge Crystallographic Data Centre via www.ccdc.cam.ac.uk (accessed on 19 December 2021).

Most beneficial LPs have been predominantly characterized from strains affiliated with the *P. fluorescens* and *P. putida* group. The chemical diversity of *Pseudomonas* LPs has been detailed in two recent reviews [1,21]. Table 1 shows the diversity of beneficial LPs and presents the discovery of similar LPs from diverse strains, countries, niches and environments. Not all LPs listed have been functionally characterized, however, the disease suppressive capacity of their producing strain(s) has been established on specific plant hosts thus indicating non-virulence. Clearly, the *P. koreensis* subgroup presents the highest diversity of LP families and individual members, including variants. This SG is characterized by at least six amphisin group members alongside the novel rhizoamide, the bananamide group comprising six variants and the cocoyamide/gacamide group. Moderate LP diversity is showcased by the *P. fluorescens* SG while the *P. protegens* SG comprises various orfamide variants A-H and the poaeamide LPs. Lastly, the *P. putida* group contains four described LP types: entolysin, putisolvin, xantholysin, WLIP and a novel 17AA LP named N8. Figure 4 shows the chemical structures of representative biocontrol LPs that have been characterized.

## 6. *Pseudomonas* LPs: Broad Spectrum Arsenals for Biological Control of Plant Pathogens

The *Pseudomonas* genus being a tremendous source of diversity, uniquely houses new strains and species [35]. The biocontrol activity displayed by these strains, corresponding LP mutants or crude as well as purified LPs has been summarized in Table 2. In this section, these active LPs will be discussed according to the taxonomic affiliation of producing strains and their respective LP groups.

### 6.1. The P. fluorescens SG: Houses the Viscosin Group, Certain Members of the Tolaasin Group and the Poaeamide Producer

Until recently, the *P. fluorescens* SG appeared to have strains producing the highest LP diversity. Besides the tolaasin group, LPs produced within this *Pseudomonas* subgroup mainly belong to the viscosin group. Members of this group comprise an oligopeptide of 9AA, differing from each other mainly by the presence of a Leu, Ile or Val at positions 4 and 9, and the 3-hydroxy-fatty acid tail length (10 to 12 carbons) [21]. Members of this group include viscosin (vis), viscosinamide (vsm), massetolide (mass), pseudodesmin (pdm), pseudophomin (psm) and the white line inducing principle (WLIP). Viscosin has been described from multiple sources; strains causing head rot of broccoli [84], marine environment [85], and the sugar beet plant phyllosphere [35,86]. Massetolide A has been isolated from strains associated with the leafy red sea algae surface [85] and from the wheat rhizosphere isolate, *P. lactis* SS101 [34]. Within the *P. fluorescens* SG, the WLIP was first reported in *P.* “*reactans*” [69] and subsequently detected in several *Pseudomonas* strains including *P.* “*reactans*” NCPPB1311 [42] *P. putida* RW10S2 (now called *P. promisalinigenes* RW10S2), a rice rhizosphere isolate [67], the biocontrol strain *P. chlororaphis* PB-St2, an isolate from sugarcane stems [69] and from the cocoyam rhizosphere isolate, NSE1 [11,68]. Two viscosinamide producers, *P. fluorescens* DR54 and *Pseudomonas* sp. A2W9.4, were isolated from sugar beet [37,87] and cocoyam rhizosphere [38], respectively. Pseudophomins A and B are produced by strain BRG100, a green foxtail rhizosphere isolate [39]. The most recently reported member of this group, pseudodesmins A and B, were isolated from *Pseudomonas* bacteria obtained from the mucus layer in the skin of the black belly salamander [88] and also recently characterized from the cocoyam rhizosphere in Cameroon [38].

Until recently, LPs belonging to the viscosin family have been most researched for biocontrol capabilities against plant pathogens including bacteria, fungi and oomycetes. In vitro assays using cell cultures, cell-free culture supernatants of *P. fluorescens* SBW25 or purified viscosin showed an efficacy in the immobilization and subsequent lysis of zoospores of *Phytophthora infestans* [35]. The biocontrol potency of SBW25 due to viscosin against *P. infestans* was further strengthened in tests involving viscosin-deficient mutants. Similar positive effects of local and induced systemic resistance in the control of *P. infestans*-mediated late blight of tomato was obtained using cell cultures and cell-free supernatants of the massetolide producer (SS101), Tn5 massetolide mutants and purified massetolide A [72]. In contrast, the massetolide production by *P. lactis* SS101 was not required to suppress the complex *Pythium* populations on apple and wheat [89]. Furthermore, a salicylic acid-dependent resistance response was successfully induced by SS101 on *Arabidopsis thaliana* against *Pseudomonas syringae* pv. *syringae* [73]. However, the probable role of massetolide in the observed induced systemic resistance (ISR) was not investigated.

Although no LP mutants have been constructed in pseudophomin, pseudodesmin and viscosinamide-producing strains, the bioactivity of these LPs has been demonstrated against Gram-positive/negative bacteria and/or plant pathogens. Pseudophomins A and B showed antifungal activity against *Leptosphaeria maculans* and *Sclerotinia sclerotiorum* [39] and were also antagonistic towards several human pathogenic Gram-positive bacteria [88].

*In vitro* tests with viscosinamide (Vsm) against *P. ultimum* and *R. solani* showed a reduction in biomass and radial growth of mycelium [14,36,70]. *In situ* application of Vsm resulted in decreased oospore formation and sclerotia formation in *P. ultimum* and *R. solani*, respectively [36,37,70,71,87]. In a more recent study, in vitro tests with varying Vsm concentrations showed that nanomolar levels caused hyphal distortion and branching of *P. myriotylum* including hyphal lysis at 1 µM [38]. Similarly, in in vitro tests against *R. solani* AG2-2, nanomolar to micromolar levels of Vsm resulted in blockage of hyphal formation, hyphal distortion and pronounced LP evasion phenotypes [38].

WLIP, a LP produced by strains belonging to the *P. fluorescens* SG, *P. chlororaphis* SG and the *P. putida* G, was found to inhibit the brown blotch disease on *Agaricus bisporus* caused by *P. tolaasii* [90]. In a related study, cell-free crude extract containing WLIP from *P.* “*reactans*” SPC 8907 inhibited the same pathogen [91]. Furthermore, WLIP production by *P.* “*reactans*” NCPPB1311 demonstrated antagonism against *Erwinia carotovora* subsp. *carotovora* (now called *Pectobacterium carotovorum*) and *Agaricus bisporus* [42]. Subsequent biological screening against fungi and bacteria indicated that WLIP is more effective against Gram-positive bacteria than Gram-negative [42]. In a separate study, tests with purified WLIP (from *P. chlororaphis* Pb-St2) did not show efficacy in antifungal tests involving *R. solani* AG2-2 and AG4 isolates [69]. In contrast, WLIP obtained from *Pseudomonas* sp. NSE1 showed excellent efficacy when tested against the same AG2-2 pathogen but using a different experimental set up [38]. Thus, in comparative studies, the need for a consistent approach when comparing compound efficacies cannot be overemphasized. Like results obtained with *R. solani* AG2-2, variable WLIP concentrations resulted in complete hyphal destruction of *P. myriotylum* [38] Moreover, Tn5 insertion WLIP mutants of *P. promysalinigenes* RW10S2 led to an antagonism against plant pathogenic *Xanthomonas* species and inhibited the growth of several Gram-positive bacteria in vitro [67].

Purified poaeamide solution caused immobilization and subsequent lysis of *Phytophthora capsici* and *P. infestans* zoospores within 1 min exposure at CMC concentrations of 50 μg/mL [41]. In contrast, cell-free culture supernatants of the WT strain did not cause these responses in the pathogens tested. When grown in direct contact with the 50 μg/mL LP solution, the inhibitory effect on dry weight mycelial biomass was observed in *P. capsici*, *R. solani* and *P. infestans* whereas a similar effect was only accomplished in *P. ultimum* at 250 μg/mL. Furthermore, an inhibitory effect was observed in the mycelial fresh weight for the same pathogens except for *P. infestans*.

The composition and length of the peptide chain of the tolaasin group ranges between 19 to 25 amino acids with the lipid tail comprising of 3-HDA or 3-hydroxyoctanoic acid (3-HOA). Purified tolaasin I from *P. tolaasii* NCPPB2192 inhibited the growth of fungi namely *Agaricus bisporus*, *Lentinus edodes*, *Pleurotus* spp. and Gram-negative bacteria belonging to the genera *Erwinia*, *Agrobacterium*, *Xanthomonas*, *Escherichia* and *Pseudomonas* [42]. Sessilins also belong to the tolaasin group of LPs but are produced by *P. sessiligenes* CMR12a, an affiliate of the *P. protegens* SG.

### 6.2. The P. koreensis SG: LP Cocktail Comprising Amphisin, Bananamide and Cocoyamide Groups

The *P. koreensis* group is a LP cocktail group comprising at least three different LP groups. In recent years, several novel LPs and derivatives have been characterized from strains situated within this subgroup. The amphisin LP group are produced within this species and comprise members including amphisin, anikasin, arthrofactin, lokisin, milkisin and tensin (Table 1). Tensin was derived from the *P. fluorescens* strain 96.578. Amphisin-, lokisin- and tensin-producing *Pseudomonas* strains demonstrated a high level of antagonism against *P. ultimum* and *R. solani* [51]. Subsequent in vitro assays using these three compounds confirmed their antagonism against these two pathogens [14,51]. Recent in vitro studies using a lokisin deletion mutant of the *P. koreensis* S150 strain showed reduced activity against *Phytophthora nicotianae* and a complete loss of inhibition against *R. solani* [49]. Additionally, purified lokisin lysed the mycelia of the cocoyam root rot pathogen, *P. myriotylum* [10]. *Pseudomonas* sp. DSS73 inhibited the root pathogenic fungus *R. solani* partially because of amphisin production [43]. A latest LP addition is rhizoamide A (formerly described as N2) which has been shown to cause hyphal lysis of *P. myriotylum* at low concentrations [10,11]. Full structural and functional analysis of rhizoamides A and derivatives B-D will be described elsewhere.

The first members of the bananamide group, bananamides I-III, were described from the banana rhizosphere isolate BW11P2 [52]. More so, a closely related LP (yet to be chemically characterized) is reportedly produced by a wheat rhizosphere isolate, *P. azadiae* SWRI103 [92]. So far, the antimicrobial and biocontrol activities of bananamides I-III have not been demonstrated. Another member of this group, bananamide D, was described from cocoyam rhizosphere isolates, COW3 and COW65 [29]. Like the first producer, COW3 produced other variants namely bananamides E, F and G. Strain COW3 suppressed cocoyam root rot disease (CRRD) in soil assays [10] while in in vitro tests, bananamides D-G inhibited the growth of *P. myriotylum* in a dose-dependent manner and further induced hyphal branching and leakage [29]. In the same study, the growth of *P. oryzae* was significantly inhibited by bananamide A, while other derivatives only displayed a mild effect. Lastly, mycophagous behaviour of bananamide D producers was observed against *P. oryzae* although it is unclear whether this is directly or indirectly due to LP production.

Cocoyamide A/Gacamide A, belonging to a new LP group, was first described from the cocoyam rhizosphere isolate *Pseudomonas* sp. COW5 [10] and from the GacA complemented *P. fluorescens* Pf0-1 strain [53]. *Pseudomonas* sp. COW5 effectively protected cocoyams from the CRRD while 10 µM of purified cocoyamide A was sufficient to cause lysis of the host-adapted cocoyam pathogen, *P. myriotylum* [10]. Purified gacamide was also described as having a moderate, narrow-spectrum antibiotic activity against clinical bacterial isolates [53].

### 6.3. The P. protegens SG: Home to Multiple Orfamide Derivatives and Sessilins

Orfamides were first extracted from *P. protegens* Pf-5 and subsequently from *P. protegens* CHA0, *P. protegens* F6 [93], *P. sessiligenes* CMR12a [59], *Pseudomonas* sp. Cab57 [94], *P. aestus* CMR5c, *P. fluorescens* Wayne1R, *Pseudomonas* spp. CMAA1215 and PH1b [56]. Multiple orfamide derivatives are produced by different strains including orfamides A-G (Table 1); strains Pf-5 and CHA0 both produce orfamides A, B and C whereas CHA0 produces an additional G derivative. More so, both CMR12a and CMR5c produce orfamides B, D, and E while the latter also produced F and G derivatives. Using the water agar–LP droplet assay, 100 µM of orfamides (A, B and G) caused hyphal branching indicative of mycelium growth inhibition in *R. solani* AG-4 HGI [56,75], contrasting negative results were obtained for the same pathogen when the agar diffusion assay method was used [79]. Orfamide B increased hyphal branching of *R. solani* AG2-1, the pathogen causing damping-off of Chinese cabbage [75]. At concentrations of 25 µM or higher, orfamides A, B and G caused zoospore lysis of *P. ultimum* and *Phytophthora porri* CBS 127099 [56]. Previous studies also showed that orfamide A and viscosin family of LPs, WLIP and viscosinamide can lyse zoospores of the oomycete pathogen, *Phytophthora ramorum* [35,71,79]. Soil assays with orfamide biosynthesis mutants revealed that orfamide B, produced by *P. sessiligenes.* CMR12a, work synergistically with phenazines and sessilins to suppress *R. solani* AG4-mediated root rot of bean, damping-off of Chinese cabbage caused by *R. solani* AG2-1 [75] and *Pythium* root rot of cocoyams [77]. Besides its efficacy against plant pathogens, orfamide A showed dose-dependent insecticidal mortality against aphids [93] and was reported to be a major determinant in the oral toxicity of Pf-5 against *Drosophila melanogaster* [95]. Additionally, experiments conducted using mutants and purified orfamide A showed that orfamide A could not elicit induced systemic resistance against the rice blast pathogen, *P. oryzae* but successfully elicited ISR against *Cochliobolus miyabeanus*, the causal pathogen of brown spot disease. Interestingly, introducing high inoculum of strain CHA0 successfully mediated ISR against *C. miyabeanus* on rice while this could not be achieved with strain CMR12a [78].

Sessilin, produced by the cocoyam rhizosphere isolate *P. sessiligenes* CMR12a, is structurally related to tolaasin and only differs from tolaasin I by one amino acid. Direct application of crude sessilin extracts resulted in vacuole formation and subsequent lysis of the mycelia of *P. myriotylum* [77]. Besides sessilin, CMR12a produces orfamide and two phenazine derivatives, PCA and phenazine-1-carboxamide (PCN). Using mutant analysis and soil assay experiments, sessilin was shown to be involved in the suppression of bean root rot due to *R. solani* AG2-2 [74], AG4 [75] and in the control of damping-off disease of cabbage caused by *R. solani* AG2-1 [75].

### 6.4. P. chlororaphis SG: Pseudodesmin, WLIP and Uncharacterized Viscosin Group LPs

The *P. chlororaphis* group comprises strains from the soil and the rhizosphere of diverse plant hosts [19]. Multiple secondary metabolites have been reported in this group [96] including the production of viscosin group lipopeptides. The sugarcane stem isolate, *P. chlororaphis* subsp. *aurantiaca*, produces WLIP but the compound did not display antifungal or antibacterial activity [69]. Viscosin group LPs have been described although their full structure and bioactivity potential is yet to be deciphered [96]. Furthermore pseudodesmin has been characterized from the *P. chlororaphis*-grouped cocoyam rhizosphere isolate, *Pseudomonas* sp. COR52 [38], thus its antimicrobial activity will be discussed here. Using a broth microdilution method, the antimicrobial activity of synthetic pseudodesmin (Pdm) was shown against six Gram-positive bacterial pathogens [97]. In this study, no antifungal effect was recorded against *Candida albicans* and *Aspergillus fumigatus*, suggesting that this compound is mainly active against Gram-positive bacteria. However, a follow up study showed the antifungal activity of this LP against the tropical cocoyam root rot pathogen, *P. myriotylum*, and the bean root rot pathogen, *R. solani* AG2-2 [38]. 100 nM and micromolar concentrations of Pdm inhibited the mycelial growth of *P. myriotylum*, effected hyphal distortions and branching, while specific Pdm concentrations (100 nM and 25 µM) resulted in a unique hyperbranching phenotype [38]. When similar concentrations were tested against *R. solani* AG2-2, multiple hyphal changes were also observed including growth inhibition, hyphal distortion, blockage and lysis.

### 6.5. P. mandelii SG, P. asplenii SG and P. corrugata SG: Thin Borderline between Pathogenic and Beneficial LPs I

In contrast to the *P. mandelii* SG, strains affiliated with the *P. asplenii* and *P. corrugata* SGs are predominantly plant pathogenic species which may also double up as plant beneficial bacteria [28]. The production of multiple LPs is a key characteristic of strains in these groups and in most cases, the complementary and synergistic roles played by the LPs in phytotoxicity, virulence or biocontrol have been reported and discussed. The antimicrobial activities of associated purified as well as crude LPs produced within these taxonomic groups have been recently summarized [28]. Here, we succinctly highlight the roles of beneficial LPs in these otherwise plant-pathogenic groups. Furthermore, we present a comparison of *Pseudomonas* strains taxonomy, the AA composition of their LPs and reported activity against three plant pathogenic classes (Figure 3).

***P. mandelii*:** Nunamycin and nunapeptin, LPs produced by *P. fluorescens* In5 were reported to be key components for the biocontrol activity of In5 [18]. Differential inhibition was exhibited by both LPs against similar plant pathogens; nunamycin inhibited mycelial growth of *R. solani* AG3 but was not effective against *Pythium aphanidermatum* whereas, with nunapeptin, the opposite scenario played out—the later pathogen was inhibited in vitro or suppressed in soil assays with tomato whereas, *R. solani* AG3 was not [18].

***P. corrugata*:** Thanamycin, brabantamide A and thanapeptin LPs are produced by *Pseudomonas* sp. SH-C52, a *R. solani*-suppressive soil isolate [60,81,98]. Experiments using mutants constructed in the thanamycin BGC and with the pure compound revealed the involvement of thanamycin in the biocontrol of SH-C52 against *Sclerotium rolfsii* on groundnut, *R. solani* on sugarbeet and the Gram-positive bacterium *B. megaterium,* but little activity against oomycete pathogens and certain Gram-negative bacteria. Brabantamide showed activity against Gram-positive bacteria such as *Staphylococcus aureus* and *Arthrobacter crystallopoietes* [99,100] and up to 50 µM was necessary to exhibit anti-oomycete activity against *P. capsici and P. infestans* [60]. However, phospholipases of the late blight pathogen, *P. infestans* were affected upon overnight co-incubation with 5 µM of brabantamide. Comparison of two Tn5 thanapeptin BGC mutants with the WT strain SH-C52 showed that thanapeptin is active against oomycetes but is not antifungal [60]. In vitro tests using purified compounds of seven thanapeptin derivatives revealed substantial differences in anti-oomycete activity such that compounds with the lowest mass had the strongest activity. For closely related LPs, syringopeptin (produced by *P. syringae* G isolates) and corpeptin (produced by *P. corrugata* SG strains), which are closely related LPs, no anti-oomycete activity has been reported [101,102].

Similar to reported LP producers in the *P. corrugata* SG, brasmycin and braspeptin LPs are produced by *Pseudomonas* sp. 11K1 [62]. A brasmycin deletion mutant lost inhibition activity against *Botryosphaeria dothidea* whereas the braspeptin mutant exhibited reduced antifungal activity. Both CLPs were not antibacterial against *Xanthomonas oryzae* RS105. Co-inoculation of purified sclerosin, produced by *P. brassicacearum* DF41 with *Sclerotinia sclerotiorum,* showed inhibition of ascospore and sclerotia germination but was not active against zoospores of *P. infestans* [61].

***P. asplenii*:** The *P. asplenii* subgroup consists of *P. fuscovaginae* (*Pfv*) and *P. asplenii* species comprising rice-infecting pathogens causing sheath rot and grain discoloration symptoms [103]. Disease surveys of rice-infected fields in tropical ecologies (Philippines) have revealed several *Pfv*-related strains which form a distinct population which differ from the type *Pfv* strains and were thus referred to as being *Pfv*-like [103]. Typically, *Pfv* and *Pfv*-like species produce syringotoxins, fuscopeptin A (FP-A) and fuscopeptin B (FP-B) which are key virulence factors in rice sheath rot infection [103,104,105,106]. Although *Pfv* and related strains are not typically biocontrol strains, the antimicrobial activity of its associated LPs have been reported. Like syringotoxin, the purified fuscopeptin inhibited the growth of *B. cinerea* and *R. pilimanae* [104].

*Pfv*-like strains *Pseudomonas* spp. COR33 and COR18 were isolated from the tropical cocoyam rhizosphere in Cameroon [10]. Similar to known *Pfv*-like strains, COR18 produces multiple LPs; a novel cyclic LP named N5, having 13AA with eight in the macrocycle (13:8) and at least a novel peptin-like LP named N7 [11]. Similarly, COR33 produces N5 but differs from typical *Pfv*-like strains since it does not produce multiple LPs. In soil assays, strain COR33 suppressed the cocoyam root rot disease caused by *P. myriotylum*. The structural and functional characterization of COR18 and COR33 LPs vis-à-vis those of *Pfv* LPs will be published shortly. In a recent review paper, LP-13 (identical to N5) was reported to be produced by *Pfv* strains in addition to fuscopeptin, syringotoxin and cryptic BGCs [28]. The clustering of COR18 (a multiple LP producer) with *Pfv*-like strains and the distinct separation of COR33 (a single LP producer) from this group provided the possibility to investigate LP evolution and function within the *P. asplenii* group. Although biocontrol assays on cocoyams or other crops are yet to be conducted using COR18, it is unlikely to be phytotoxic on cocoyam since it was isolated from the healthy cocoyam rhizosphere.

### 6.6. P. syringae G: Thin Borderline between Pathogenic and Beneficial LPs II

Popular biocontrol *P. syringae* group isolates include the *P. syringae* pv. *syringae* B359 (alternatively named B497 or HS191), *P. syringae* pv. *syringae* B301 and *P. syringae* ESC-10 and ESC-11. Strains ESC-10 and ESC-11 effectively protected both lemons and oranges against green and blue molds caused by *Penicillium digitatum* and *Penicillium italicum*, respectively. The superior efficacy of *P. syringae* strain ESC-10 in controlling postharvest pathogens on citrus crops led to the development of Bio-Save, an EPA registered product containing ESC-10 as its active ingredient [83]. The application of Bio-Save reduced green and blue mold incidence on lemons and oranges by 87.9% and 58.6%, respectively. ESC-11 is used for the control of postharvest pathogens on apple and pear. Both ESC-10 and ESC-11 are commercialized as Bio-SAVE 10 and Bio-SAVE 11, respectively [83]. However, the biocontrol capacity of these strains is not due to LPs.

Syringomycin, syringotoxin and syringopeptin are phytotoxins produced by *P. syringae* which generally induce necrosis [107]. These LPs are known to act as virulence factors and facilitate disease severity when produced. The peptide portion of syringopeptin contains either 22 (SP22) or 25 (SP25) amino acids that are mainly hydrophobic. SP25 is produced by strains isolated from infected millet (B359) and citrus (B427), while SP22 is produced by a *P. syringae* isolate from pear (B301) and variants by strains obtained from different hosts. Besides enhancing virulence of the producing pathogens, purified syringomycin E (SR-E), syringotoxin (ST) and two forms of syringopeptin (SP_22_-A and SP_25_-A) exhibited antimicrobial activity against specific Gram-positive bacteria and fungi. Although the growth of *Bacillus megaterium* was inhibited by 1.56 µM SP_22_-A and 3.12 µM SP_25_-A, SR-E and ST had no effect [82]. With respect to fungi, the yeast *Rhodotorula pilimanae* was most sensitive to SR-E but was also inhibited by SP_22_-A and SP_25_-A. The plant pathogenic ascomycete *B. cinerea* was inhibited by 1.6 µM SP_25_-A, 12.5 µM SP_22_-A, 18.7 µM SR-E and 25 µM ST [82]. Despite having similar spore-forming characteristics in artificial membrane bilayer assays, these different *P. syringae* toxins have distinct antimicrobial activities.

### 6.7. P. putida G: Beneficial LPs with Broad-Spectrum Targets

Within the *P. fluorescens* lineage, the *P. putida* group is the second largest, containing about 69 species that occupy diverse ecological niches [6,7,27]. Besides their role in plant growth promotion and soil remediation, *P. putida* strains have functions in direct antagonism and ISR against plant pathogens [108,109]. So far, four main lipopeptides belonging to diverse families, have been described from strains within this group [20,110] (Table 1). Among the *P. putida* LPs is the WLIP whose bioactivity has been described in this review as a member of the viscosin group, within the *P. fluorescens* SG.

Following the discovery of the WLIP, putisolvins I and II have only been described from *P. putida* PCL 1445, isolated from a site polluted with polycyclic aromatic hydrocarbons [111] and *P. putida* 267, isolated from the black pepper rhizosphere [65]. More recently, putisolvins III, IV and V were described from cocoyam rhizosphere isolates *Pseudomonas* spp. COR19, COR55, NNC7, WCU_60, WCU_64 [10,11] and in *P. putida* LMG 11722^T^, the type strain of *P. fulva* [110]. Additionally, the prominent plant -growth promoting and -disease suppressing strain, *P. capeferrum* WCS358, also produces putisolvin but this has not appear to be a determinant in its plant growth promoting and/or its induced resistance abilities [110,112,113]. Although in vitro tests with partially purified putisolvins obtained from *P. putida* 267 resulted in the lysis of zoospores of *Phytophthora capsici* within 90 s, a putisolvin mutant in the same strain did not result in the loss of biocontrol against pre- and post-emergence damping-off of cucumber caused by *P. capsici* [65]. Furthermore, *Pseudomonas* sp. COR55 suppressed the cocoyam root rot pathogen whereas purified putisolvin III from the same strain inhibited mycelial growth of *P. myriotylum* and caused hyphal branching [10]. Putisolvin producers were dominant in the soils of Cameroon and Nigeria and were described as being conducive to the cocoyam root rot disease caused by *P. myriotylum* [11].

Entolysins A and B were first described from *P. entomophilia* L48T, a strain considered to be a natural pathogen of *Drosophila* [114]. Using an entolysin mutant (*etlC*), this LP was shown to be important for the haemolytic and swarming capacity of L48T but not involved in biocontrol observed in a cucumber-*P. ultimum* pathosystem [66]. The cocoyam rhizosphere isolate, *Pseudomonas* sp. COR5, also produces entolysin B and provided 100% protection against *Pythium* root rot on cocoyams. In the same study, 10 µM of purified entolysin B inhibited the mycelial growth of *P. myriotylum* in a dose-dependent manner [10]. Several entolysin producers have been characterized from the healthy cocoyam rhizosphere in Cameroon [10,11].

The banana rhizosphere isolate, *P. mosselii* BW11M1, produces xantholysins A-D [63]. Besides the role of xantholysin in swarming and biofilm formation, analysis of xantholysin mutants showed both antifungal and antibacterial activities of this compound. During in vitro experiments, xantholysin showed toxicity to diverse *Xanthomonas* spp. including broad antifungal activity against an ascomycete (*Botrytis cinerea*) and a basidiomycete (*R. solani*), among others. More so, xantholysin producers were prominently found in the healthy cocoyam rhizosphere of *Pythium* root rot suppressive soils in Cameroon andosols [10]. The xantholysin producer, *Pseudomonas* sp. COR51 suppressed *Pythium* root rot disease on cocoyams while 10 µM of purified xantholysin A showed anti-oomycete activity by inhibiting hyphal growth and inducing hyphal branching of *P. myriotylum* [10]. Based on in vitro tests with pure compounds/mutants xantholysin appears to have broad-spectrum antifungal activity against the major fungal classes [63]. Xantholysin A production was recently described in *P. xantholysinigenes* RW9S1A^T^ [110].

The latest addition to the *P. putida* LPs is the novel N8, a 17AA LP which contains 8AA in the macrocycle. N8 is produced by the cocoyam rhizosphere isolate, *Pseudomonas* sp. COR35 [10,11]. Full chemical, biosynthetic and functional characterization of this LP will be elucidated shortly. In soil assays, strain COR35 effectively protected cocoyam against the root rot disease caused by *P. myriotylum* [10]. Like other *P. putida* LPs, 10 µM of purified N8 inhibited the mycelial growth of the oomycete pathogen, *P. myriotylum* [10].

### 6.8. Mapping Strain Taxonomy to LP Chemistry and Antimicrobial Activity

**Figure 5:**
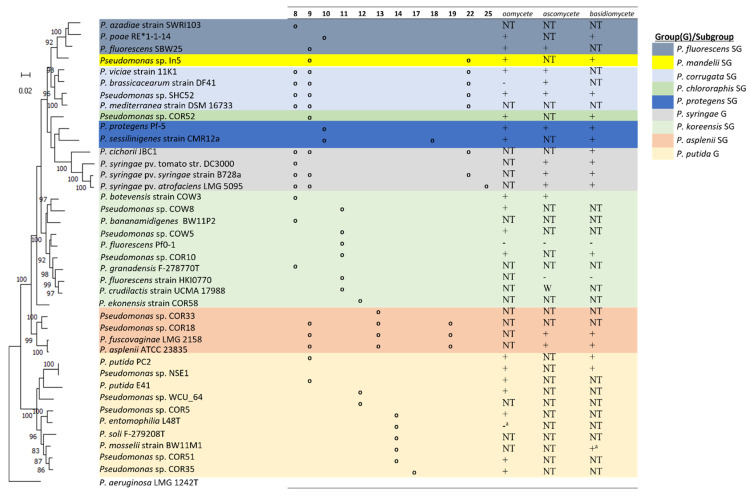
Comparison of Lipopeptide Amino Acid (AA) Composition and In Vitro Biological Activity within the *Pseudomonas* Genus. Within each group/subgroups, representative strains producing unique LPs with available genomes on NCBI were selected. Sequences of 16S rRNA and housekeeping genes (*gyrB*, *rpoB* and *rpoD*) were used; maximum likelihood tree, GTR+G+I model (MEGA-X) [115]. Bootstrap values were calculated based on 1000 replications. *P. aeruginosa* was used as an outgroup. Strains within the *P. fluorescens*, *P. chlororaphis*, *P. protegens*, *P. koreensis*, and *P. putida* (sub)groups have single LPs with 8, 9, 10, 11, 12, 14 or 17AA. An exception is strain CMR12a (*P. protegens* SG) which produces two LPs (10 and 18AA). Multiple LPs are produced by strains affiliated with the *P. mandelii* (9 and 22AA), *P. corrugata* SG (8, 9 and 22AA), *P. syringae* G (8, 9, and 22/25 AA) and *P. asplenii* SG (8, 13, and 19AA). Biological activity conducted in in vitro tests using purified compounds are shown. +: LP active; -: LP inactive; NT: LP not tested; -^a^: LP mutant tested.

To map *Pseudomonas* LP-producing groups to AA composition of LPs and the antimicrobial efficacies of purified LPs, we generated a concatenated Multilocus Sequence Analysis phylogenetic tree from 16S rRNA and housekeeping genes (*gyrB*, *rpoB* and *rpoD*) sequences derived from the draft/whole genomes of each strain (Figure 5). Strains producing three LPs can be of two types: Type I (8, 9 and 22/25 AA) comprising the *P. corrugata* and *P. syringae* SG candidates while the Type II group (9, 13, 19AA) are affiliated with the *P. asplenii* SG. Dual LP producers are situated in the *P. mandelii* SG (strain In5) and the *P. protegens* SG (strain CMR12a). In principle, strains situated in other SGs (*P. fluorescens* SG, *P. koreensis* SG, *P. protegens* SG (except CMR12a)) and the *P. putida* G produce a single LP. Clearly, the antimicrobial efficacy of purified LPs against plant pathogens have been under-researched. Figure 5 shows the antimicrobial efficacy (or absence) of a particular LP when only one is produced by a strain. In cases of multiple LP production, a positive activity indicates the efficacy of at least one LP against the test pathogen class. The specific pathogens tested for strains in Figure 5 has been mentioned in Table 2. In most cases, no LP has been tested against representative members of the three pathogen classes except for strain SH-C52 (Figure 5).

## 7. *Pseudomonas* LPs: Emerging Broad-Spectrum Arsenals in Plant–Pathogen and Microbe–Microbe Interactions

### 7.1. LP-Mediated Induced Systemic Resistance (ISR)

Induced systemic resistance is a phenomenon in which bacteria with biocontrol potential enhance the plant defense against pathogen invasion and insect herbivores [116]. ISR mirrors the systemic acquired resistance (SAR) triggered upon pathogen perception or recognition by cell surface receptors and cytoplasmatic receptors. Cell surface or pattern recognition receptors (PRR) recognize molecules containing pathogen- or microbe-associated patterns (PAMPs or MAMPs). Examples of bacterial MAMPs are the 22 AA flagellin peptide flg22, lipopolysaccharides and peptidoglycan, among others [117]. In mammals, Toll-like receptors (TLRs) are the most-studied PRRs, and at least 13 TLRs have been identified that are involved in the recognition of several different MAMPs [118,119]. Toll-like receptors are a family of type I transmembrane pattern recognition receptors (PRRs) that sense invading pathogens or endogenous damage signals and subsequently initiate the innate and adaptive immune response. TLR4 for example, recognizes lipopolysaccharides (LPSs) from Gram-negative bacteria, whereas TLR5 is specific in the recognition of bacterial flagellin [119,120]. DOTAP, a cationic lipid widely used as a liposomal transfection reagent, has been identified as a strong activator of the innate immunity system mainly in animal cells and recently, in plants [121]. This cationic lipid which is recognized by TLR4, triggered a plant defense response in the model plant *A. thaliana*, evidenced by callose deposition, reactive oxygen species production, plant cell death, proteomic analysis and against the virulent bacterial pathogen, *Pseudomonas syringae* pv. *tomato* DC3000 (Pst) [121]. In plants, the best-characterized PRRs belong to the receptor-like kinases (RLKs) or the receptor-like proteins (RLPs) [118].

MAMPs binding results in early immune-related events in sensitive cells such as ion fluxes, the phosphorylation cascade and the oxidative burst. This is accompanied by antimicrobial and phytoalexin (secondary metabolite) stimulation coupled with induction of cell wall reinforcement [122]. On the other hand, the pattern triggered immunity (PTI) activated in the plant can be toned down by certain pathogens via the injection of protein effectors into the host cells, thereby blocking the immune response in the plant. To counteract this, plants produce resistance (R) proteins leading to effector-triggered immunity (ETI). Both PTI and ETI can result in SAR [31]. As with pathogenic bacteria, beneficials can be detected by the plant receptor machinery [123]. They also need to evade or suppress PTI in order to establish a cooperation with their host plant [124,125]. Subsequently, beneficial bacteria successfully and efficiently colonize their host thus enabling secretion of metabolites such as lipopeptides which in turn may result in multiple benefits including ISR. Unlike MAMPs, lipopeptides appear not to be perceived by cell surface receptors, but interact with the lipid bilayer fraction of plant plasma membranes in a process that is poorly understood. Molecular mechanisms underlying ISR by lipopeptides have been recently reviewed [30,31].

Some *Pseudomonas* LPs demonstrate ISR activity against diverse foliar pathogens of monocots and dicots. First, using massetolide mutants in the *P. lactis* strain SS101, it was shown that massetolide A displayed ISR elicitation in the control of *P. infestans* in tomato plants [72]. Subsequently, strain SS101 enhanced resistance in *Arabidopsis thaliana* against several plant pathogens including *P. syringae* pv *tomato* (*Pst*), although the role of massetolide in this interaction was not investigated [60].

Besides *P. sessiligenes* CMR12a’s capacity for direct antagonism against *R. solani* via an interplay between sessilin, orfamide and phenazines [59,75], this strain demonstrated ISR against *R. solani* and *Cochliobolus miyabeanus* on common bean and rice, respectively [76,78] (Table 2). In monocots, such as rice, orfamide successfully induced resistance towards *C. miyabeanus* whereas crude CLP extracts of WLIP, lokisin and entolysin, induced resistance toward *M. oryzae* [68,76,78]. On the other hand, WLIP-producing strains induced resistance against the rice blast disease whereas induction was absent in treatments with WLIP mutants [68]. Although the bananamide producer *Pseudomonas* sp. COW3 induced resistance against rice blast, crude extracts of bananamide D from the same strain were not effective in soil assays although they successfully blocked appressoria formation by *M. oryzae* during in vitro experiments [68]. In contrast, root inoculations with orfamide producers *P. protegens* CHA0 and *Pseudomonas* sp. CMR5c did not induce resistance against rice blast [78,126]. Similarly, rice plants drenched with purified orfamides were not effective in resistance induction [78] although purified orfamides A, B and G actively inhibited appressoria formation and reduced the number of susceptible lesions on rice [56].

**Figure 6:**
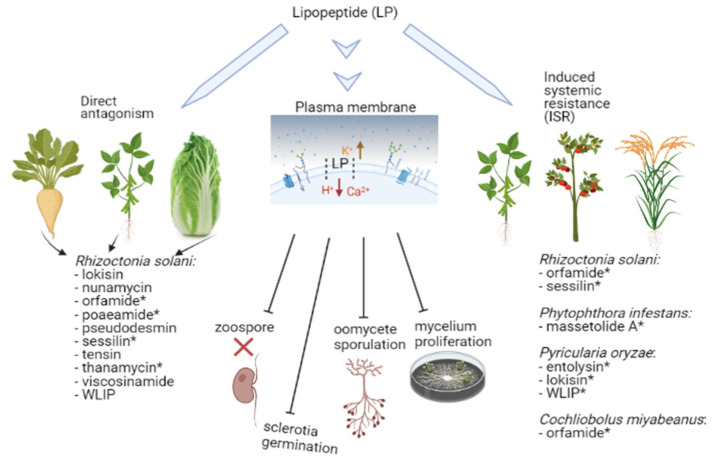
Scheme Showing the Membrane Interaction of LPs Together with their Bioactivity Routes via Direct Antagonism and Induced Systemic Resistance (ISR). LPs perturb the membrane barrier resulting in an influx of H^+^ and Ca^2+^ together with an efflux of K^+^. In in vitro tests, LPs successfully lyse zoospores and block sclerotia germination, oomycete sporulation/germination and mycelium proliferation. In direct antagonism, diverse LPs mediate *R. solani* suppression on bean, Chinese cabbage and in *in vitro* tests. For ISR, LPs induce resistance against *R. solani* on bean, *Phytophthora infestans* on tomato, *Pyricularia oryzae* on rice and *Cochliobolus miyabeanus* on rice. Asterisks (*) indicate LPs that were tested *in planta* while the other LPs were tested in the absence of a plant host.

The origin of LP-mediated antimicrobial activity has been attributed to membrane perturbation, specifically, pore formation [21]. Several LPs have been shown to permeabilize model membranes probably via transmembrane pore formation (Figure 6) [42,127]. Subsequent to pore formation, the pH gradient across the membrane is thought to collapse via increase of the H^+^ and Ca^2+^ ions influx including the efflux of K^+^ ions (Figure 6) [128,129]. Consequently, calcium-mediated signaling pathways are induced leading to cell death. For example, the cell membrane is implicated as the primary site of tolaasin action. Tolaasin can disrupt the membranes of fungal, bacterial, plant and animal cells [130], forming ion channels in planar lipid bilayers and this membrane conductance activity was highly dependent on toxin concentration. Similar to other membrane-active peptides, LPs may cause cellular disruption by protein pore formation in the membranes [127]. Previously, the natural decanoic (C10) pseudodesmin (pdm) was shown to be more active against a panel of Gram-positive bacteria strains in comparison with synthesized pdm C4 to C8 and C12 to C14. In a recent study, the membrane-permeabilizing activity of natural pdm was compared with those of the aforementioned synthetic variants [131]. By employing the fluorescence lifetime leakage assay (a technique used to assess calcein release from liposomes), it was shown that antagonistic concentrations and chain length dependence correlate with liposome leakage and antimicrobial activity. The mechanism of action of *Pseudomonas* LPs have been summarized [21] and clearly, accelerated biophysical studies are required to further expand our knowledge regarding LP modes of action.

### 7.2. Microbial Competition: Bacterial Mycophagy and White Line-in-Agar Interaction

Bacterial mycophagy refers to a set of phenotypic behaviors which enable the bacteria to grow at the expense of living fungal tissue [132]. Bananamides D-G producers, *Pseudomonas* spp. COW3 and COW65, belonging to the *P. koreensis* SG, demonstrated mycophagous behavior in separate co-incubation experiments with the rice blast pathogen *M. oryzae* and the cocoyam root rot pathogen, *P. myriotylum* [29]. Since COW3 and COW65 were isolated from the cocoyam rhizosphere of the *Pythium* suppressive Boteva soil [10], it is plausible that these strains can contribute to soil suppressiveness and attendant plant health via competition with pathogenic organisms such as *P. myriotylum*. Recent studies suggest that microbial competition among soil saprophytes may have resulted in the general suppressiveness identified in the Boteva soil. Pseudomonads isolated from Boteva have a high LP diversity (n = 11) which may be driven by antagonist–antagonist interactions. Besides, of these 11 diverse LPs, strains producing five unique LPs (cocoyamide, bananamide D, entolysin, WLIP and N8) showed a white line-in-agar phenotype in interaction with the CMR12a mutant which produced sessilin [11,59]. The in vitro interaction between the pathogenic *P. tolaasii* and another *Pseudomonas* bacterium, referred to as ‘*P. reactans*’ produced a sharply defined white line precipitate [42,133]. The formation of a white line precipitate by two co-inoculated LP producers and a beehive of this activity in a disease suppressive andosol [11] gives an indication of the role of LPs in microbial defense and warding off niche competitors belonging to similar or different genera.

## 8. Conclusions and Future Perspectives

In this review, we chronicled LP-producing strains across *Pseudomonas* groups, their LPs and biological activity demonstrated against diverse plant pathogens classes (Table 1 and Table 2). In general, there is a good correlation between taxonomy and LP type produced. *Pseudomonas* biocontrol strains and their respective LPs are largely associated with strains situated in the *P. fluorescens* and *P. putida* group. Within the *P. fluorescens* group, diverse beneficial LPs are produced by strains belonging to the *P. fluorescens*, *P. chlororaphis*, *P. koreensis*, *P. mandelii*, *P. corrugata* and *P. asplenii* subgroups with *P. koreensis* and *P. fluorescens* SG recording the largest LP diversity. However, there are exceptions to taxonomy-LP correlations due to convergent evolution or horizontal gene transfer. For example, WLIP is produced by strains of the *P. fluorescens* SG, *P. chlororaphis* SG and *P. putida* G. However, the BGCs encoding this LP are considerably different thereby indicating that these gene cluster evolved via convergent evolution. Similarly, pseudodesmins are produced by both *P. tolaasii* and *P. chlororaphis* SGs. The possibility for strains to obtain LP BGCs by horizontal gene transfer is exemplified by the sessilin (tolaasin-like) BGCs which are situated on a genomic island in the strain CMR12a. Thus, the tight linkage of LPs within specific taxonomic groups irrespective of the plant host reinforces the idea of a rapidly evolving system that develops new molecules by randomly shuffling and swapping domains and modules. A recent study showed that the phylogeny of PleB, the MacB-like transporter driving export of *Pseudomonas* LPs, correlated strongly with LP chemical diversity [110]. Thus, the possibility of matching chemistry to taxonomy provides a starting point for LP predictions once the phylogenic affiliation of a biosurfactant-producing *Pseudomonas* strain has been deciphered.

Another aspect of research that is limiting is the investigation of how specific *Pseudomonas* taxonomy and LP diversity impacts soil and plant health. Recent studies report the presence of *P. koreensis* group strains encoding diverse LPs in the cocoyam rhizosphere of a *Pythium* suppressive soil [11]. It will be interesting to obtain insights into which taxonomic assemblage and metabolite(s) are recruited for the inhibition of unique pathogen classes. Specifically, which LPs are better suited to inhibit oomycetes, basidiomycetes and ascomycetes. The possibility to prescribe a LP(s) ‘package’ to suppress specific pathogen classes will significantly contribute to sustainable plant protection.

With respect to biological control activity screening, diverse LP-producing strains, LP mutants and crude as well as purified LPs have been tested against plant pathogens. For example, with the *P. putida* G, all associated LPs were effective against oomycetes in in vitro assays (Table 1). Purified putisolvin and entolysin were effective against oomycetes in in vitro tests but both LPs were not involved in biocontrol of oomycetes in soil assays using mutant strains (Table 2). Although xantholysin mutants have been generated, they have only been used in in vitro tests and demonstrated moderate to strong activity against ascomycetes [63]; whereas purified compounds were only tested against *Xanthomonas* spp. WLIP-producing wild type; and mutant strains have been utilized in both in vitro and ISR experiments hence shedding more light on their biological function (Table 2). In order to obtain clear insights into LP activity, adopting a systematic approach to test the biocontrol efficacy of LP-producing strains versus mutants against diverse pathogens and on different plant hosts has become necessary. The effect of substrate characteristics and inoculum preparation strategies should be optimized both for direct antagonism assays and soil drench/foliar experiments aimed at eliciting ISR. Besides, high-throughput techniques should be employed to test molecule efficacy against Gram-positive and Gram-negative bacteria, fungi and nematodes.

Moreover, only the structure–function activity of orfamides A-G [56] and bananamides D-G [29] were preliminarily studied. However, an elaborate and effective approach which employs LP synthesis of both variable fatty acid length and amino acid composition was employed for pseudodesmin structure–function activity experiments [134] Clearly, this method needs to be explored further to elucidate LP structure–activity relationships. Lastly, the determination of Minimal Inhibitory Concentrations (MICs) and/or IC_50_ values for classes of LPs will enable the mapping of LP structure to function giving room for the development of sustainable and targeted LP-based crop protection strategies.

In spite of intensive research on *Pseudomonas* biocontrol, only a few *Pseudomonas* strains are commercialized. In general, Pseudomonads display inconsistent field performance, which can be attributed to poor adaptation to environmental conditions and host-specific responses to microorganisms [19]. Specifically, LP formulation is still suboptimal and production costs must be drastically reduced to enable commercial field usage especially when high amounts of compounds are necessary for direct antagonism or elicitation of systemic resistance against plant pathogens. Moreover, the costly and arduous registration procedure of new microbial inoculants particularly in Europe, hampers advancement in this field.

## Figures and Tables

**Table 1:** Taxonomy of LP-producing Biocontrol Pseudomonads, their corresponding Molecules and Origin.

Taxonomy	Biocontrol Strains	Host/Origin	Country	LP Family	LP	Reference
*P. fluorescens* SG	SS101	Wheat rhizosphere	Netherlands	Viscosin	Massetolide	[34]
	SBW25	Sugarbeet phyllosphere	UK		Viscosin	[35]
	DR54	Sugarbeet rhizosphere	Denmark		Viscosinamide	[36,37]
	A2W4.9, U2W1.5	White cocoyam rhizosphere	Nigeria		Viscosinamide	[38]
	BRG100	Green foxtail rhizosphere	Canada		Pseudophomin	[39]
	RE*1-1-14	Internal part of soybean roots	Germany		Poaemide	[40,41]
	NCPPB1311	Cultivated mushrooms	UK		WLIP	[42]
*P. koreensis* SG	DSS73	Sugarbeet rhizosphere	Denmark	Amphisin	Amphisin	[14,43]
	HKI0770	Forest soil	Forest soil		Anikasin	[44,45]
	CTS17	Sugarbeet rhizosphere	Denmark		Hodersin	[14,46]
	DSS41	Sugarbeet rhizosphere	Denmark		Lokisin	[47]
	2.74	Tomato hydroponics	Sweden		Lokisin	[48]
	S150	Tobacco rhizosphere	China		Lokisin	[49]
	COR10	Red cocoyam rhizosphere	Cameroon		Lokisin	[10]
	UCMA 17988	Raw bulk tank milk	France		Milkisin	[50]
	COW8	White cocoyam rhizosphere	Cameroon		Rhizoamide (N2—11:7) †	[11]
	96.578	Sugarbeet rhizosphere	Denmark		Tensin	[37,51]
	BW11P2	Banana rhizoplane	Sri Lanka	Bananamide	Bananamide I, II, III	[12,52]
	COW3, COW65	White cocoyam rhizosphere	Cameroon		Bananamide D, E, F, G	[10,29]
	COW5	White cocoyam rhizosphere	Cameroon	Cocoyamide	Cocoyamide A	[10]
	Pf0-1	Loam soil	USA		Gacamide A	[53,54]
*P. protegens* SG	CHA0	Tobacco roots	Switzerland	Orfamide	Orfamide	[55,56]
	Pf-5	Cotton rhizosphere	USA		Orfamide	[57,58]
	CMR5c	Red cocoyam rhizosphere	Cameroon		Orfamide	[56]
	CMR12a	Red cocoyam rhizosphere	Cameroon		Orfamide, Sessilin	[59]
*P. chlororaphis* SG	COR52	Red cocoyam rhizosphere	Cameroon	Viscosin	Pseudodesmin	[38]
*P. mandelii* SG	In5	Suppressive potato soil	Greenland	Syringomycin	Nunamycin	[18]
	In5	Suppressive potato soil	Greenland	Syringopeptin	Nunapeptin	[18]
*P. corrugata* SG	SH-C52	Sugarbeet rhizosphere	Netherlands	Syringomycin	Thanamycin	[60]
	DF41	Canola root	Canada		Thanamycin -var1	[28,61]
	11K1	Bean rhizosphere	China		Brasmycin	[62]
	SH-C52	Sugarbeet rhizosphere	Netherlands	Syringopeptin	Thanapeptin	[60]
	DF41	Canola root	Canada		Sclerosin	[61]
	11K1	Bean rhizosphere	China		Braspeptin	[62]
*P. putida* G	BW11M1	Banana rhizoplane	Sri Lanka	Xantholysin	Xantholysin	[12,63]
	COR51	Red cocoyam rhizosphere	Cameroon		Xantholysin	[10]
	BS011	Rice rhizosphere	China		Xantholysin	[64]
	267	Black pepper	Vietnam	Putisolvin	Putisolvin I, II	[65]
	COR55	Red cocoyam rhizosphere	Cameroon		Putisolvin III, IV, V	[10,11]
	L48	Fly	Guadeloupe	Entolysin	Entolysin A, B	[66]
	COR5	Red cocoyam rhizosphere	Cameroon		Entolysin B	[10]
	RW10S2	Rice rhizosphere	Sri Lanka	Viscosin	WLIP	[67]
	COW10	White cocoyam rhizosphere	Cameroon		WLIP	[10]
	NSE1	White cocoyam rhizosphere	Nigeria		WLIP	[68]
	COR35	Red cocoyam rhizosphere	Cameroon	Unclassified	N8 (17:8) †	[11]
*P. asplenii* SG	COR33	Red cocoyam rhizosphere	Cameroon	Unclassified	N5 (13:8) †	[11]
	COR18	Red cocoyam rhizosphere	Cameroon		N5 (13:8), N7 †, Mycin LP †	[11]
Novel U2 SG	COR58	Red cocoyam rhizosphere	Cameroon	Unclassified	N4 (12:10) †	[10,11]

† novel LPs.

**Table 2:** LPs Tested Against Plant Pathogens using In Vitro, Soil and Foliar Assays.

Strain and Taxonomy	Plant	Pathogen	Lipopeptide	Experimental Setup	Method *	Reference
*P. fluorescens* SG						
*P. fluorescens* DR54	Sugar beet	*Pythium ultimum*	Viscosinamide	soil, *in vitro*	Pure	[14,36,70]
	Sugar beet	*Rhizoctonia solani*	Viscosinamide	soil, *in vitro*	Pure	[14,36,71]
*Pseudomonas* sp. A2W4.9	-	*Pythium myriotylum*	Viscosinamide	*in vitro*	Pure	[38]
	-	*Rhizoctonia solani* AG2-2	Viscosinamide	*in vitro*	Pure	[38]
*P. lactis* SS101	Tomato	*Phytophthora infestans*	Massetolide A	soil assay, foliar	Mutant, pure	[72]
	Arabidopsis	*Pseudomonas syringae* pv. *tomato*	Massetolide A	soil assay (ISR), *in vitro*	Mutant	[73]
	Hyacinth bulbs	*Pythium intermedium*, *Pythium* spp., *Phytophthora infestans*, *Albugo candida*	Massetolide A	*in vitro*	Mutant	[34]
*P. fluorescens* SBW25	-	*Phytophthora infestans*	Viscosin	*in vitro*	Mutant	[35]
*P. fluorescens* BRG100	-	*Leptosphaeria maculans*, *Sclerotinia sclerotiorum*	Pseudophomin A and B	*in vitro*	Pure	[39]
*Pseudomonas* sp. COR52	-	*Pythium myriotylum*	Pseudodesmin	*in vitro*	Pure	[38]
	-	*Rhizoctonia solani* AG2-2	Pseudodesmin	*in vitro*	Pure	[38]
*P. poae* RE *1-1-14	-	*Phytophthora capsici*, *Phytophthora infestans*	Poaeamide	*in vitro*	Pure	[41]
		*Pythium ultimum*, *Rhizoctonia solani*		*in vitro*	Pure	[41]
*P. reactans* NCPPB1311	-	*Erwinia carotovora* subsp. *carotovora*, *Agaricus bisporus*	WLIP	*in vitro*	Pure	[42]
*P. reactans*	-	*Pseudomonas tolaasii*	WLIP	*in vitro*, mushroom cap	Pure	[42]
*P. tolaasii* NCPPB2192	-	*Escherichia coli*, *Erwinia*, *Agrobacterium*, *Pseudomonas*, *Xanthomonas*, *Pleurotus* spp., *Agaricus bisporus*	Tolaasin 1	*in vitro*	Pure	[42]
*P. protegens* SG						
*P. sessiligenes* CMR12a	Bean	*Rhizoctonia solani* AG2-2, AG4	Sessilin	soil assay	Mutant	[74,75]
		*Rhizoctonia solani* AG2-2 (web blight)	Sessilin	soil assay (ISR)	Mutant, crude extract	[76]
		*Rhizoctonia solani* AG2-1, AG4	Sessilin	*in vitro*	Crude extract	[75]
	Rice	*Pyricularia oryzae*	Sessilin	soil assay (ISR)	Mutants	[76]
	Chinese cabbage	*Rhizoctonia solani* AG2-1	Sessilin	soil assay	Mutant	[75]
	Cocoyam	*Pythium myriotylum*	Sessilin	soil assay	Mutant	[77]
		*Pythium myriotylum*	Sessilin	*in vitro*	Crude extract	[77]
	Bean	*Rhizoctonia solani* AG4	Orfamide	soil assay	Mutant	[75]
	Bean	*Rhizoctonia solani* AG2-2 (web blight)	Orfamide B	soil assay (ISR)	Mutant, pure	[76]
	-	*Rhizoctonia solani* AG2-1, AG4	Orfamide B	*in vitro*	Mutant, pure	[75]
	Chinese cabbage	*Rhizoctonia solani* AG4	Orfamide	soil assay	Mutant	[75]
	Cocoyam	*Pythium myriotylum*	Orfamide	soil assay	Mutant	[77]
	-	*Pythium myriotylum*	Orfamide B	in vitro	Pure	[77]
	Rice	*Pyricularia oryzae*	Orfamide	soil assay (ISR)	Mutants	[76]
	Rice	*Pyricularia oryzae*	Orfamide A	soil assay (ISR)	Pure	[78]
*P. protegens* CHA0	Rice	*Cochliobolus miyabeanus*	Orfamide A	soil assay (ISR)	Mutant	[78]
	-	*Phytophthora porri*, *Pythium ultimum*		*in vitro*	Pure	[56]
	-	*Rhizoctonia solani* AG4		*in vitro*	Pure	[56]
	Rice	*Cochliobolus miyabeanus*		soil drench (ISR)	Pure	[78]
	-	*Phytophthora ramorum*		*in vitro*	Pure	[79]
*P. aestus* CMR5c	-	*Rhizoctonia solani* AG4	Orfamide B	*in vitro*	Pure	[56]
	-	*Pyricularia oryzae*		*in vitro*	Pure	[56]
	-	*Phytophthora porri*, *Pythium ultimum*		*in vitro*	Pure	[56]
	-	*Pyricularia oryzae*	Orfamide G	*in vitro*	Pure	[56]
	-	*Rhizoctonia solani* AG4		*in vitro*	Pure	[56]
	-	*Phytophthora porri*, *Pythium ultimum*		*in vitro*	Pure	[56]
*P. chlororaphis* SG *Pseudomonas* sp. COR52	-	*Pythium myriotylum*	Pseudodesmin	*in vitro*	Pure	[38]
	-	*Rhizoctonia solani*	Pseudodesmin	*in vitro*	Pure	[38]
*P. koreensis* SG						
*P. botevensis* COW3	-	*Pythium myriotylum*	Bananamide D, E, F, G	*in vitro*	Pure	[29]
		*Pyricularia oryzae*	Bananamide D, E, F, G	*in vitro*	Pure	[29]
	Rice	*Pyricularia oryzae*	Bananamide D, E, F, G	soil assay (ISR)	Crude extract	[68]
*Pseudomonas* sp. COW5	-	*Pythium myriotylum*	Cocoyamide	*in vitro*	Pure	[10]
*P. fluorescens* Pf0-1	-	*Pseudomonas syringae*, *Erwinia amylovora*	Gacamide	*in vitro*	Pure	[53]
*Pseudomonas* sp. DSS73	-	*Rhizoctonia solani*, *Pythium ultimum*	Amphisin	*in vitro*	Mutant, pure	[14,43]
*P. fluorescens* HKI0770		*Polysphondylium violaceum*	Anikasin	*in vitro*	Pure	[44]
*Pseudomonas* sp. COR10	-	*Pythium myriotylum*	Lokisin	*in vitro*	Pure	[10]
	Rice	*Pyricularia oryzae*	Lokisin	soil assay (ISR)	Crude extract	[68]
*Pseudomonas* sp. UCMA 17988		*Penicillium expansum*	Milkisin	*in vitro*	Pure	[50]
*Pseudomonas* sp. COW8	-	*Pythium myriotylum*	N2 (Rhizoamide (11:7))	*in vitro*	Pure	[10,11]
*Pseudomonas* sp. DSS41	-	*Rhizoctonia solani*, *Pythium ultimum*	Lokisin	*in vitro*	Pure	[14]
*Pseudomonas* sp. 2.74	Tomato	*Pythium ultimum*	Lokisin	hydroponic assay	Crude extract	[48]
*Pseudomonas* sp. 96.578	-	*Rhizoctonia solani*	Tensin	*in vitro*	Pure	[46,51]
*Pseudomonas* sp.	-	*Rhizoctonia solani*, *Pythium ultimum*	Hodersin	*in vitro*	Pure	[14]
*P. corrugata* SG						
*Pseudomonas* sp. SH-C52	Groundnut	*Sclerotium rolfsii*	Thanamycin	nethouse and field	Mutant	[80]
	-	*Botrytis cinerea*, *Geotrichum* sp., *Rhizoctonia solani*	Thanamycin	*in vitro*	Mutant	[60]
	Sugar beet	*Rhizoctonia solani*	Thanamycin	soil assay	Mutant	[81]
	-	*Rhizoctonia solani*	Thanamycin	*in vitro*	Mutant	[81]
		*Phytophthora infestans*, *Pythium ultimum*	Thanapeptin	*in vitro*	Mutant	[60]
*P. brassicacearum* DF41	Canola	*Sclerotinia sclerotiorum*	Sclerosin	soil assay, foliar spray	Mutant	[61,80]
*P. brassicacearum* 11K1		*Botryosphaeria dothidea*	Brasmycin	*in vitro*		[62]
		*Botryosphaeria dothidea*	Braspeptin	*in vitro*		[62]
*P. mandelii* SG						
*P. fluorescens* In5		*Rhizoctonia solani*	Nunamycin	*in vitro*	Mutant	[18]
		*Pythium aphanidermatum*	Nunapeptin	*in vitro*	Mutant	[18]
*P. syringae* G						
*P. syringae* pv. *syringae* B359 (B427)	-	*Botrytis cinerea*, *Rhodotorula* *pilimanae*	Syringotoxin	*in vitro*	Pure	[82]
*P. syringae* pv. *syringae* B301	-	*Botrytis cinerea*, *Geotrichum candidum*	Syringomycin E	*in vitro*	Pure	[82]
*P. syringae* ESC-10 and ESC-11	Lemon	*Penicillium digitatum*		*in vitro, in planta*	Pure	[83]
*P. syringae* pv. *syringae* B359 (B427)		*Botrytis cinerea*, *Geotrichum candidum*	Syringopeptin (SP_22_-A, SP_25_-A)	*in vitro*	Pure	[82]
*P. putida* G						
*P. entomophilia* L48	Cucumber	*Pythium ultimum*	Entolysin	soil assay	Mutant	[66]
*Pseudomonas* sp. COR5	-	*Pythium myriotylum*	Entolysin	*in vitro*	Pure	[10]
	Rice	*Pyricularia oryzae*	Entolysin	soil assay (ISR)	Crude extract	[68]
*P. putida* 267		*Phytophthora capsici*	Putisolvin	*in vitro*	Mutant	[13]
*Pseudomonas* sp. COR55	*-*	*Pythium myriotylum*	Putisolvin	*in vitro*	Pure	[10]
*Pseudomononas* sp. NSE1	-	*Pythium myriotylum*	WLIP	*in vitro*	Pure	[11]
	-	*Rhizoctonia solani* AG2-2	WLIP	*in vitro*	Pure	[11]
*P. promisalinigenes* RW10S2	-	*Xanthomonas* sp.	WLIP	*in vitro*	Mutant	[67]
	Rice	*Pyricularia oryzae*	WLIP	soil assay (ISR)	Mutant analysis, Crude extract	[68]
*P. mosselii* BW11M1	-	*Xanthomonas* spp., *Rhizoctonia solani*, *Botrytis cinerea*	Xantholysin	*in vitro*	Mutant	[63]
*P. mosselii* BS011	Rice	*Pyricularia oryzae*	Xantholysin	soil assay (ISR)	Crude extract	[64]
				*in vitro*	Crude extract	[64]
	-	*Pythium myriotylum*	Xantholysin A	*in vitro*	Pure	
*Pseudomonas* sp. COR51	Rice	*Pyricularia oryzae*	Xantholysin	soil assay (ISR)	Crude extract	[68]
*Pseudomonas* sp. COR35	-	*Pythium myriotylum*	N8 (17:8)	*in vitro*	Pure	[10]

* Pure: refers to purified LP molecules; Rows highlighted in green depict pure LPs/crude LPs that were inactive or mutant strains that still showed activity, indicating that the LP was not involved.

## Supplementary Materials

The following supporting information can be downloaded, Table S1: Comparative Genome Blast Analysis of Reference genome (Pf0-1, accession number: NC_007492) CDS with CDS of Lipopeptides Biosynthetic Gene Clusters (BGCs) in Query genomes. Blast Hits of BGC genes/products are highlighted blue. BGC-coding genes are flanked by upstream and/or downstream by transcriptional regulators and transport genes.

## Appendix A

**Table A1:** Representative Lipopeptide-Producing *Pseudomonas* Genomes Used to Generate the Comparative Genome Blast Atlas, with Strains Affiliated to Different Taxonomic Groups and Subgroups.

Strain	Lipopeptide	Accesssion Number	Reference
*P. azadiae* sp. *nov.* SWRI103	Uncharacterized	NZ_JAHSTY010000001	[92]
*P. fluorescens* SBW25	Viscosin	NC_012660.1	[35]
*P. lactis* SS101	Massetolide	NZ_CM001513.1	[135]
*P. poae* RE*1-1-14	Poaeamide	NC_020209.1	[41]
*P. tolaasii* strain 2192T	Tolaasiin	NZ_CP020369.1	[136]
*P. fluorescens* Pf0-1	Gacamide	NC_007492.2	[53]
*P. fluorescens* MS82	Putative Bananamide producer	NZ_CP028826.1	[29,137]
*P. kribbensis* 46-2 ^T^	Bananamide-like	NZ_CP029608.1	[138]
*P. granadensis* LMG 27940	MDN-066 (Bananamide-like)	NZ_LT629778.1	[139,140]
*P. chlororaphis* PBSt-2	WLIP	CP027716.1	[69]
*P. chlororaphis* DSM 21509	Viscosin group	NZ_LT629761.1	[96]
*P. chlororaphis* Lzh-T5	Viscosin group	NZ_CP025309.1	[38]
*P. protegens* Cab57	Orfamide	NZ_AP014522.1	[94]
*P. protegens* Pf-5	Orfamide	NC_004129.6	[79]
*P. protegens* CHA0	Orfamide	NC_021237.1	[56]
*P. sessilinigenes* sp. *nov.* CMR12a	Orfamide and Sessilin	NZ_CP077074.1	[59]
*P. fuscovaginae* LMG 2158T	Fuscopeptin, syringotoxin	NZ_LT629972.1	[28]
*P. asplenii* ATCC 23835	Fuscopeptin, syringostatin	NZ_LT629777.1	[28]
*P. mandelii* LMG 21607 ^T^ = LMG 2210	Uncharacterized	NZ_LT629796.1	[26]
*P. brassicacearum* DF41	Sclerosin, Thanamycin-var1	NZ_CP007410.1	[61,141]
*P. mediterranea* DSM 16733 ^T^	Thanamycin, Peptin 22-var1	NZ_LT629790.1	[28]
*P. viciae* 11K1	Brasmycin, Braspeptin	NZ_CP035088.1	[62]
*P. syringae* B728a	Syringomycin, Syringopeptin SP22, Syringafactin	NC_007005.1	[28,142]
*P. syringae* B301D	Syringomycin, Syringopeptin SP22, Syringafactin	NZ_CP005969.1	[143,144]
*P. syringae* pv. *syringae* HS191	Syringomycin, Syringopeptin SP25, Syringafactin	NZ_CP006256.1	[143,144]
*P. syringae* pv. *tomato* DC3000	Syringafactin	NC_004578.1	[145]
*P. cichorii* JBC1	Pseudomycin, Cichopeptin, cichofactin	NZ_CP007039.1	[1,146]
*P. entomophilia* L48 ^T^	Entolysin	NC_008027.1	[114]
*P. putida* PC2	Putative WLIP producer	NZ_CP011789.1	[68]
*P. soli* SJ10	Xantholysin-like	NZ_CP009365.1	[147]
*P. putida* E41	Putative Putisolvin producer	NZ_CP024085.1	[11]
*P. mosselii* BS011	Xantholysin	CP023299.1	[64]

gold: *P. fluorescens* SG; light orange: *P. koreensis* SG; light blue: *P. chlororaphis* SG; light green: *P. protegens* SG; grey: *P. asplenii SG*; red: *P. mandelii* SG; dark green: *P. corrugata* SG; yellow: *P. syringae* G; and blue: *P. putida* G. ^T^: denotes type strain.
