# Supplementary material for: Pseudomonas Lipopeptide-Mediated Biocontrol: Chemotaxonomy and Biological Activity
Source: Molecules. 2022 Jan 7;27(2):372. doi: 10.3390/molecules27020372 (PMC8777863; doi:10.3390/molecules27020372)
Supplement: Supplementary file 1 [file molecules-27-00372-s001.zip › molecules-1512444-supplementary.pdf]

Table S1. Comparative Genome Blast Analysis of Reference genome (Pf0-1, accession number: NC_007492) CDS with CDS of Lipopeptides Biosynthetic Gene Clusters (BGCs) in Query genomes. Blast Hits of BGC genes/products are highlighted blue. BGC-coding genes are flanked by upstream and/or downstream by transcriptional regulators and transport genes.

Sequence ID	Hit Sequence ID	Hit File	Hit Gene	Hit Product	Percent Identity
NC_007492	NZ_CM001513	P._lactis_SS101.gb		copper chaperone PCu(A)C	87.04
NC_007492	NZ_LT629778	P._granadensis_LMG_27940.gb		copper chaperone PCu(A)C	83.51
NC_007492	NC_020209	P._poae_RE1_1_14.gb		copper chaperone PCu(A)C	83.41
NC_007492	NZ_CP023299	P._mosselii_BS011.gb		copper chaperone PCu(A)C	80.62
NC_007492	NZ_CP020369	P._tolaasii_2192T.gb		copper chaperone PCu(A)C	84.97
NC_007492	NZ_CP011789	P._putida_PC2.gb		copper chaperone PCu(A)C	82.48
NC_007492	CT573326	P._entomophilia_L48.gb		conserved hypothetical protein. putative signal peptide	84.40
NC_007492	NZ_CP029608	P._kribbensis_46_2.gb		copper chaperone PCu(A)C	88.12
NC_007492	NZ_CP006256	P._syringae_pv._syringae_HS191.gb		copper chaperone PCu(A)C	80.60
NC_007492	NC_012660	P._fluorescens_SBW25.gb		copper chaperone PCu(A)C	83.01
NC_007492	CP028826	P._fluorescens_MS82.gb		copper chaperone PCu(A)C	97.48
NC_007492	CP027716	P._chlororaphis_PBSt_2.gb		Cytochrome oxidase biogenesis protein Sco1/SenC/PrrC	83.00
NC_007492	NC_007005	P._syringae_pv._syringae_B728a.gb		SCO family protein	82.51
NC_007492	CP024085	P._putida_E41.gb		SCO family protein	82.39
NC_007492	CT573326	P._entomophilia_L48.gb		conserved hypothetical protein. putative Sco1/SenC family protein	80.07
NC_007492	NZ_CP007039	P._cichorii_JBC1.gb		SCO family protein	81.46
NC_007492	NC_021237	P._protegens_CHA0.gb		SCO family protein	82.78
NC_007492	NZ_CP005969	P._syringae_pv._syringae_B301D.gb		SCO family protein	82.80
NC_007492	NZ_LT629796	P._mandelii_LMG_21607.gb		SCO family protein	81.89
NC_007492	NZ_CP077074	P._sessilinigenes_CMR12a.gb		SCO family protein	81.94
NC_007492	NC_004129	P._protegens_Pf_5.gb		SCO family protein	83.25
NC_007492	AE016853	P._syringae_pv._tomato_str._DC3000.gb		Sco1/SenC family protein	81.61
NC_007492	LT629761	P._chlororaphis_DSM_21509.gb		protein SCO1/2	83.50
NC_007492	NZ_CP023299	P._mosselii_BS011.gb		SCO family protein	85.00
NC_007492	NZ_CP009365	P._soli_SJ10.gb		SCO family protein	84.11
NC_007492	NZ_CP006256	P._syringae_pv._syringae_HS191.gb		SCO family protein	80.85
NC_007492	CP028826	P._fluorescens_MS82.gb		SCO family protein	97.85
NC_007492	NZ_CP011789	P._putida_PC2.gb		SCO family protein	81.52
NC_007492	NZ_CP029608	P._kribbensis_46_2.gb		SCO family protein	93.56
NC_007492	NZ_CP020369	P._tolaasii_2192T.gb		SCO family protein	80.24
NC_007492	NC_020209	P._poae_RE1_1_14.gb		SCO family protein	80.00
NC_007492	LT629761	P._chlororaphis_DSM_21509.gb		protein SCO1/2	83.50
NC_007492	NZ_LT629777	P._asplenii_ATCC_23835.gb		SCO family protein	84.10
NC_007492	NZ_LT629972	P._fuscovaginae_LMG_2158.gb		SCO family protein	83.66
NC_007492	NZ_CP005969	P._syringae_pv._syringae_B301D.gb		SCO family protein	82.80
NC_007492	NC_012660	P._fluorescens_SBW25.gb		SCO family protein	81.82
NC_007492	NZ_AP014522	P._protegens_Cab57.gb		SCO family protein	82.78

NC_007492	NZ_LT629778	P._granadensis_LMG_27940.gb		SCO family protein	86.47
NC_007492	NC_021237	P._protegens_CHA0.gb		efflux transporter outer membrane subunit	81.29
NC_007492	NZ_CP023299	P._mosselii_BS011.gb		AdeC/AdeK/OprM family multidrug efflux complex outer membrane factor	81.00
NC_007492	NZ_LT629778	P._granadensis_LMG_27940.gb		efflux transporter outer membrane subunit	82.67
NC_007492	NC_004129	P._protegens_Pf_5.gb		efflux transporter outer membrane subunit	81.40
NC_007492	NC_004129	P._protegens_Pf_5.gb		efflux RND transporter outer membrane subunit EmhC	80.67
NC_007492	NC_012660	P._fluorescens_SBW25.gb		AdeC/AdeK/OprM family multidrug efflux complex outer membrane factor	83.65
NC_007492	NZ_CP007410	P._brassicacearum_DF41.gb	adeC	AdeC/AdeK/OprM family multidrug efflux complex outer membrane factor	83.02
NC_007492	AE016853	P._syringae_pv._tomato_str._DC3000.gb		outer membrane efflux protein	83.80
NC_007492	NZ_LT629790	P._mediterranea_DSM_16733.gb	adeC	AdeC/AdeK/OprM family multidrug efflux complex outer membrane factor	85.56
NC_007492	NC_007005	P._syringae_pv._syringae_B728a.gb		AdeC/AdeK/OprM family multidrug efflux complex outer membrane factor	80.34
NC_007492	CP024085	P._putida_E41.gb		multidrug transporter	81.93
NC_007492	LT629761	P._chlororaphis_DSM_21509.gb		outer membrane protein, multidrug efflux system	80.04
NC_007492	NZ_LT629796	P._mandelii_LMG_21607.gb	emhC	efflux RND transporter outer membrane subunit EmhC	83.58
NC_007492	CP024085	P._putida_E41.gb		multidrug transporter	82.55
NC_007492	NZ_LT629778	P._granadensis_LMG_27940.gb	emhC	efflux RND transporter outer membrane subunit EmhC	81.76
NC_007492	NZ_CM001513	P._lactis_SS101.gb		efflux transporter outer membrane subunit	85.88
NC_007492	NC_021237	P._protegens_CHA0.gb	adeC	AdeC/AdeK/OprM family multidrug efflux complex outer membrane factor	82.74
NC_007492	NZ_CP035088	P._viciae_11K1.gb	adeC	AdeC/AdeK/OprM family multidrug efflux complex outer membrane factor	83.84
NC_007492	NZ_CP077074	P._sessilinigenes_CMR12a.gb		efflux transporter outer membrane subunit	82.23
NC_007492	NZ_CP020369	P._tolaasii_2192T.gb	adeC	AdeC/AdeK/OprM family multidrug efflux complex outer membrane factor	82.99
NC_007492	CP027716	P._chlororaphis_PBSt_2.gb		Multidrug efflux system, outer membrane factor lipoprotein OprM	81.02
NC_007492	NZ_JAHSTY010000001	P._azadiae_SWRI103.gb		efflux transporter outer membrane subunit	80.31
NC_007492	NZ_CP007039	P._cichorii_JBC1.gb		AdeC/AdeK/OprM family multidrug efflux complex outer membrane factor	80.76
NC_007492	NZ_CP009365	P._soli_SJ10.gb		AdeC/AdeK/OprM family multidrug efflux complex outer membrane factor	81.82
NC_007492	NZ_CP035088	P._viciae_11K1.gb	adeC	AdeC/AdeK/OprM family multidrug efflux complex outer membrane factor	81.60
NC_007492	NZ_CP005969	P._syringae_pv._syringae_B301D.gb	adeC	AdeC/AdeK/OprM family multidrug efflux complex outer membrane factor	83.74
NC_007492	NZ_CM001513	P._lactis_SS101.gb		AdeC/AdeK/OprM family multidrug efflux complex outer membrane factor	80.65
NC_007492	LT629761	P._chlororaphis_DSM_21509.gb		outer membrane protein, multidrug efflux system	80.04
NC_007492	NZ_AP014522	P._protegens_Cab57.gb		efflux transporter outer membrane subunit	81.40
NC_007492	NZ_CP020369	P._tolaasii_2192T.gb	adeC	AdeC/AdeK/OprM family multidrug efflux complex outer membrane factor	80.00
NC_007492	NZ_CP006256	P._syringae_pv._syringae_HS191.gb	adeC	AdeC/AdeK/OprM family multidrug efflux complex outer membrane factor	81.01
NC_007492	NC_007005	P._syringae_pv._syringae_B728a.gb	adeC	AdeC/AdeK/OprM family multidrug efflux complex outer membrane factor	82.93
NC_007492	CP028826	P._fluorescens_MS82.gb		multidrug transporter	94.20
NC_007492	NZ_CP029608	P._kribbensis_46_2.gb	adeC	efflux RND transporter outer membrane subunit EmhC	80.86
NC_007492	NZ_CP007410	P._brassicacearum_DF41.gb	adeC	AdeC/AdeK/OprM family multidrug efflux complex outer membrane factor	83.29
NC_007492	NZ_LT629790	P._mediterranea_DSM_16733.gb	adeC	AdeC/AdeK/OprM family multidrug efflux complex outer membrane factor	83.05
NC_007492	CT573326	P._entomophilia_L48.gb	ttgC	multidrug/solvent efflux outer membrane protein TtgC, RND family	80.71
NC_007492	NZ_CP011789	P._putida_PC2.gb		AdeC/AdeK/OprM family multidrug efflux complex outer membrane factor	83.91
NC_007492	NZ_CP029608	P._kribbensis_46_2.gb		efflux transporter outer membrane subunit	86.45
NC_007492	CT573326	P._entomophilia_L48.gb	oprM	Outer membrane protein oprM precursor	81.82

NC_007492	NZ_AP014522	P._protegens_Cab57.gb	adeC	AdeC/AdeK/OprM family multidrug efflux complex outer membrane factor	82.54
NC_007492	NZ_CP011789	P._putida_PC2.gb		efflux transporter outer membrane subunit	81.56
NC_007492	CP028826	P._fluorescens_MS82.gb		AdeC/AdeK/OprM family multidrug efflux complex outer membrane factor	81.15
NC_007492	NZ_CP006256	P._syringae_pv._syringae_HS191.gb		AdeC/AdeK/OprM family multidrug efflux complex outer membrane factor	81.95
NC_007492	NZ_CP005969	P._syringae_pv._syringae_B301D.gb	adeC	AdeC/AdeK/OprM family multidrug efflux complex outer membrane factor	83.74
NC_007492	NZ_CP077074	P._sessilinigenes_CMR12a.gb	adeC	AdeC/AdeK/OprM family multidrug efflux complex outer membrane factor	80.67
NC_007492	NC_020209	P._poae_RE1_1_14.gb		LuxR family transcriptional regulator	83.10
NC_007492	NC_012660	P._fluorescens_SBW25.gb		LuxR family transcriptional regulator	83.89
NC_007492	NZ_CP035088	P._viciae_11K1.gb		helix-turn-helix transcriptional regulator	81.76
NC_007492	NZ_CP007410	P._brassicacearum_DF41.gb		LuxR family transcriptional regulator	82.39
NC_007492	NZ_CP023299	P._mosselii_BS011.gb		LuxR C-terminal-related transcriptional regulator	85.45
NC_007492	NZ_CP011789	P._putida_PC2.gb		helix-turn-helix transcriptional regulator	83.02
NC_007492	CP028826	P._fluorescens_MS82.gb		helix-turn-helix transcriptional regulator	92.96
NC_007492	CP027716	P._chlororaphis_PBSt_2.gb		Transcriptional regulator, LuxR family	88.48
NC_007492	NZ_LT629777	P._asplenii_ATCC_23835.gb		LuxR family transcriptional regulator	82.79
NC_007492	NZ_AP014522	P._protegens_Cab57.gb		helix-turn-helix transcriptional regulator	84.85
NC_007492	NZ_LT629790	P._mediterranea_DSM_16733.gb		helix-turn-helix transcriptional regulator	83.65
NC_007492	NZ_LT629777	P._asplenii_ATCC_23835.gb		helix-turn-helix transcriptional regulator	82.76
NC_007492	NZ_LT629972	P._fuscovaginae_LMG_2158.gb		helix-turn-helix transcriptional regulator	82.76
NC_007492	CP024085	P._putida_E41.gb		helix-turn-helix transcriptional regulator	82.39
NC_007492	NZ_CP029608	P._kribbensis_46_2.gb		LuxR family transcriptional regulator	85.91
NC_007492	NC_021237	P._protegens_CHA0.gb		helix-turn-helix transcriptional regulator	83.98
NC_007492	NZ_CP009365	P._soli_SJ10.gb		LuxR C-terminal-related transcriptional regulator	84.00
NC_007492	NZ_CM001513	P._lactis_SS101.gb		LuxR family transcriptional regulator	85.21
NC_007492	NZ_CP020369	P._tolaasii_2192T.gb		helix-turn-helix transcriptional regulator	80.21
NC_007492	LT629761	P._chlororaphis_DSM_21509.gb		transcriptional regulator, LuxR family	90.05
NC_007492	NZ_JAHSTY010000001	P._azadiae_SWRI103.gb		LuxR C-terminal-related transcriptional regulator	80.00
NC_007492	LT629761	P._chlororaphis_DSM_21509.gb		transcriptional regulator, LuxR family	90.05
NC_007492	NC_004129	P._protegens_Pf_5.gb		helix-turn-helix transcriptional regulator	83.98
NC_007492	NZ_LT629972	P._fuscovaginae_LMG_2158.gb		LuxR family transcriptional regulator	83.61
NC_007492	NZ_CP077074	P._sessilinigenes_CMR12a.gb		helix-turn-helix transcriptional regulator	84.36
NC_007492	NZ_CP035088	P._viciae_11K1.gb		non-ribosomal peptide synthase/polyketide synthase	82.24
NC_007492	NZ_CP005969	P._syringae_pv._syringae_B301D.gb		amino acid adenylation domain-containing protein	86.16
NC_007492	NZ_CP005969	P._syringae_pv._syringae_B301D.gb		non-ribosomal peptide synthetase	83.54
NC_007492	NZ_CP006256	P._syringae_pv._syringae_HS191.gb		non-ribosomal peptide synthase/polyketide synthase	83.23
NC_007492	NZ_CM001513	P._lactis_SS101.gb		non-ribosomal peptide synthetase	81.76
NC_007492	NZ_CP006256	P._syringae_pv._syringae_HS191.gb	sypA	syringopeptin non-ribosomal peptide synthetase SypA	82.50
NC_007492	NZ_CP007410	P._brassicacearum_DF41.gb		non-ribosomal peptide synthetase	86.00
NC_007492	NZ_LT629972	P._fuscovaginae_LMG_2158.gb		non-ribosomal peptide synthetase	83.93
NC_007492	NZ_CP006256	P._syringae_pv._syringae_HS191.gb		non-ribosomal peptide synthetase	87.65
NC_007492	NZ_LT629972	P._fuscovaginae_LMG_2158.gb		non-ribosomal peptide synthetase	81.40

NC_007492	AE016853	P._syringae_pv._tomato_str._DC3000.gb	syfA	non-ribosomal peptide synthetase SyfA	82.58
NC_007492	NZ_CP020369	P._tolaasii_2192T.gb		non-ribosomal peptide synthetase	81.68
NC_007492	LT629761	P._chlororaphis_DSM_21509.gb		arthrofactin-type cyclic lipopeptide synthetase A	85.25
NC_007492	NC_007005	P._syringae_pv._syringae_B728a.gb	sypB	syringopeptin non-ribosomal peptide synthetase SypB	80.00
NC_007492	NZ_CP020369	P._tolaasii_2192T.gb		amino acid adenylation domain-containing protein	83.82
NC_007492	NC_004129	P._protegens_Pf_5.gb		non-ribosomal peptide synthetase	82.91
NC_007492	NZ_CP005969	P._syringae_pv._syringae_B301D.gb		amino acid adenylation domain-containing protein	86.16
NC_007492	CP027716	P._chlororaphis_PBSt_2.gb		Siderophore biosynthesis non-ribosomal peptide synthetase	81.46
NC_007492	NZ_CP023299	P._mosselii_BS011.gb		amino acid adenylation domain-containing protein	85.83
NC_007492	NZ_CP005969	P._syringae_pv._syringae_B301D.gb	sypA	syringopeptin non-ribosomal peptide synthetase SypA	81.65
NC_007492	NC_021237	P._protegens_CHA0.gb		non-ribosomal peptide synthetase	82.91
NC_007492	NZ_CP005969	P._syringae_pv._syringae_B301D.gb		amino acid adenylation domain-containing protein	80.09
NC_007492	NC_007005	P._syringae_pv._syringae_B728a.gb	sypA	syringopeptin non-ribosomal peptide synthetase SypA	82.44
NC_007492	NZ_LT629777	P._asplenii_ATCC_23835.gb		non-ribosomal peptide synthetase	85.96
NC_007492	NZ_CP020369	P._tolaasii_2192T.gb		amino acid adenylation domain-containing protein	86.57
NC_007492	NZ_CP035088	P._viciae_11K1.gb		non-ribosomal peptide synthetase	82.79
NC_007492	NZ_AP014522	P._protegens_Cab57.gb	ofaB	orfamide A non-ribosomal peptide synthetase OfaB	81.87
NC_007492	NZ_JAHSTY010000001	P._azadiae_SWRI103.gb		amino acid adenylation domain-containing protein	87.18
NC_007492	LT629761	P._chlororaphis_DSM_21509.gb		arthrofactin-type cyclic lipopeptide synthetase C	81.75
NC_007492	NZ_AP014522	P._protegens_Cab57.gb	ofaA	orfamide A non-ribosomal peptide synthetase OfaA	85.80
NC_007492	NZ_CP005969	P._syringae_pv._syringae_B301D.gb	sypA	syringopeptin non-ribosomal peptide synthetase SypA	81.65
NC_007492	NZ_CP009365	P._soli_SJ10.gb		non-ribosomal peptide synthetase	84.35
NC_007492	NZ_CP005969	P._syringae_pv._syringae_B301D.gb		non-ribosomal peptide synthetase	82.68
NC_007492	LT629761	P._chlororaphis_DSM_21509.gb		arthrofactin-type cyclic lipopeptide synthetase A	85.25
NC_007492	NZ_CP077074	P._sessilinigenes_CMR12a.gb		amino acid adenylation domain-containing protein	81.63
NC_007492	CT573326	P._entomophilia_L48.gb		putative non-ribosomal peptide synthetase	83.52
NC_007492	NZ_LT629777	P._asplenii_ATCC_23835.gb		non-ribosomal peptide synthetase	84.62
NC_007492	NZ_CP007410	P._brassicacearum_DF41.gb		non-ribosomal peptide synthetase	81.65
NC_007492	NZ_LT629777	P._asplenii_ATCC_23835.gb		non-ribosomal peptide synthase/polyketide synthase	80.81
NC_007492	NZ_CP011789	P._putida_PC2.gb		non-ribosomal peptide synthetase	82.04
NC_007492	NZ_CP005969	P._syringae_pv._syringae_B301D.gb	sypB	syringopeptin non-ribosomal peptide synthetase SypB	83.47
NC_007492	CT573326	P._entomophilia_L48.gb		putative non ribosomal peptide synthetase	81.48
NC_007492	NC_007005	P._syringae_pv._syringae_B728a.gb		non-ribosomal peptide synthetase	82.24
NC_007492	NC_007005	P._syringae_pv._syringae_B728a.gb	sypC	syringopeptin non-ribosomal peptide synthetase SypC	87.13
NC_007492	NZ_CP005969	P._syringae_pv._syringae_B301D.gb		non-ribosomal peptide synthase/polyketide synthase	82.24
NC_007492	NZ_CP009365	P._soli_SJ10.gb		non-ribosomal peptide synthetase	81.40
NC_007492	NZ_CP006256	P._syringae_pv._syringae_HS191.gb		amino acid adenylation domain-containing protein	80.60
NC_007492	CT573326	P._entomophilia_L48.gb		putative non-ribosomal peptide synthetase	81.43
NC_007492	NZ_LT629972	P._fuscovaginae_LMG_2158.gb		amino acid adenylation domain-containing protein	81.32
NC_007492	CP028826	P._fluorescens_MS82.gb		non-ribosomal peptide synthetase	82.91
NC_007492	NC_007005	P._syringae_pv._syringae_B728a.gb		non-ribosomal peptide synthetase	84.81

NC_007492	NZ_CP029608	P._kribbensis_46_2.gb		amino acid adenylation domain-containing protein	86.00
NC_007492	NZ_LT629777	P._asplenii_ATCC_23835.gb		non-ribosomal peptide synthetase	87.34
NC_007492	NZ_LT629778	P._granadensis_LMG_27940.gb		amino acid adenylation domain-containing protein	83.18
NC_007492	NZ_CP005969	P._syringae_pv._syringae_B301D.gb	sypC	syringopeptin non-ribosomal peptide synthetase SypC	87.13
NC_007492	NZ_LT629790	P._mediterranea_DSM_16733.gb		non-ribosomal peptide synthetase	85.71
NC_007492	NZ_LT629972	P._fuscovaginae_LMG_2158.gb		non-ribosomal peptide synthetase	84.31
NC_007492	NZ_AP014522	P._protegens_Cab57.gb		non-ribosomal peptide synthetase	82.91
NC_007492	NZ_CP020369	P._tolaasii_2192T.gb		amino acid adenylation domain-containing protein	80.70
NC_007492	NZ_CP020369	P._tolaasii_2192T.gb		amino acid adenylation domain-containing protein	80.92
NC_007492	NZ_CP029608	P._kribbensis_46_2.gb		non-ribosomal peptide synthetase	83.33
NC_007492	NZ_CP007039	P._cichorii_JBC1.gb		non-ribosomal peptide synthase/polyketide synthase	84.89
NC_007492	NZ_CP006256	P._syringae_pv._syringae_HS191.gb		non-ribosomal peptide synthase	84.18
NC_007492	NC_004129	P._protegens_Pf_5.gb	ofaC	orfamide A non-ribosomal peptide synthetase OfaC	86.00
NC_007492	LT629761	P._chlororaphis_DSM_21509.gb		arthrofactin-type cyclic lipopeptide synthetase B	84.40
NC_007492	NC_020209	P._poae_RE1_1_14.gb		non-ribosomal peptide synthetase	84.29
NC_007492	NZ_CP007410	P._brassicacearum_DF41.gb		non-ribosomal peptide synthetase	86.13
NC_007492	CP024085	P._putida_E41.gb		non-ribosomal peptide synthetase	84.07
NC_007492	NZ_CP011789	P._putida_PC2.gb		non-ribosomal peptide synthetase	80.41
NC_007492	NZ_CP029608	P._kribbensis_46_2.gb		amino acid adenylation domain-containing protein	89.17
NC_007492	NZ_CP020369	P._tolaasii_2192T.gb		amino acid adenylation domain-containing protein	84.06
NC_007492	NZ_LT629778	P._granadensis_LMG_27940.gb		non-ribosomal peptide synthetase	83.76
NC_007492	NZ_LT629777	P._asplenii_ATCC_23835.gb		amino acid adenylation domain-containing protein	82.05
NC_007492	NZ_CP005969	P._syringae_pv._syringae_B301D.gb	sypC	syringopeptin non-ribosomal peptide synthetase SypC	87.13
NC_007492	NZ_CP023299	P._mosselii_BS011.gb		non-ribosomal peptide synthetase	85.19
NC_007492	NZ_CP020369	P._tolaasii_2192T.gb		non-ribosomal peptide synthetase	84.09
NC_007492	NZ_CM001513	P._lactis_SS101.gb		non-ribosomal peptide synthetase	85.44
NC_007492	CP028826	P._fluorescens_MS82.gb		hypothetical protein	81.01
NC_007492	NZ_CP006256	P._syringae_pv._syringae_HS191.gb		non-ribosomal peptide synthetase	85.83
NC_007492	NZ_CP023299	P._mosselii_BS011.gb		non-ribosomal peptide synthetase	82.01
NC_007492	NC_007005	P._syringae_pv._syringae_B728a.gb		amino acid adenylation domain-containing protein	80.53
NC_007492	LT629761	P._chlororaphis_DSM_21509.gb		arthrofactin-type cyclic lipopeptide synthetase B	84.40
NC_007492	NZ_LT629790	P._mediterranea_DSM_16733.gb		amino acid adenylation domain-containing protein	85.00
NC_007492	NZ_LT629790	P._mediterranea_DSM_16733.gb		non-ribosomal peptide synthetase	82.93
NC_007492	NZ_CP077074	P._sessilinigenes_CMR12a.gb		amino acid adenylation domain-containing protein	86.59
NC_007492	NC_021237	P._protegens_CHA0.gb	ofaC	orfamide A non-ribosomal peptide synthetase OfaC	85.00
NC_007492	NZ_CP011789	P._putida_PC2.gb		amino acid adenylation domain-containing protein	83.20
NC_007492	NC_012660	P._fluorescens_SBW25.gb		non-ribosomal peptide synthetase	85.00
NC_007492	NC_021237	P._protegens_CHA0.gb	ofaB	orfamide A non-ribosomal peptide synthetase OfaB	82.76
NC_007492	NZ_CP006256	P._syringae_pv._syringae_HS191.gb	sypC	syringopeptin non-ribosomal peptide synthetase SypC	82.69
NC_007492	NC_004129	P._protegens_Pf_5.gb	ofaA	orfamide A non-ribosomal peptide synthetase OfaA	85.21
NC_007492	CT573326	P._entomophilia_L48.gb		putative non-ribosomal peptide synthetase, terminal component	81.19

NC_007492	NZ_CM001513	P._lactis_SS101.gb		non-ribosomal peptide synthetase	81.71
NC_007492	NZ_CP007039	P._cichorii_JBC1.gb		non-ribosomal peptide synthetase	86.09
NC_007492	NZ_CP007039	P._cichorii_JBC1.gb		non-ribosomal peptide synthetase	82.81
NC_007492	NZ_CP005969	P._syringae_pv._syringae_B301D.gb		amino acid adenylation domain-containing protein	80.09
NC_007492	NZ_LT629777	P._asplenii_ATCC_23835.gb		non-ribosomal peptide synthetase	81.01
NC_007492	NZ_CP005969	P._syringae_pv._syringae_B301D.gb		non-ribosomal peptide synthetase	83.54
NC_007492	CP024085	P._putida_E41.gb		non-ribosomal peptide synthetase	84.02
NC_007492	NZ_CP009365	P._soli_SJ10.gb		non-ribosomal peptide synthetase	86.31
NC_007492	NZ_CP005969	P._syringae_pv._syringae_B301D.gb	sypB	syringopeptin non-ribosomal peptide synthetase SypB	83.47
NC_007492	NZ_LT629972	P._fuscovaginae_LMG_2158.gb		non-ribosomal peptide synthetase	87.34
NC_007492	NC_012660	P._fluorescens_SBW25.gb		non-ribosomal peptide synthetase	80.34
NC_007492	NZ_LT629972	P._fuscovaginae_LMG_2158.gb		non-ribosomal peptide synthetase	81.51
NC_007492	NZ_CP035088	P._viciae_11K1.gb		amino acid adenylation domain-containing protein	81.94
NC_007492	AE016853	P._syringae_pv._tomato_str._DC3000.gb		non-ribosomal peptide synthetase, terminal component	82.89
NC_007492	NC_021237	P._protegens_CHA0.gb	ofaA	orfamide A non-ribosomal peptide synthetase OfaA	85.80
NC_007492	NZ_JAHSTY010000001	P._azadiae_SWRI103.gb		amino acid adenylation domain-containing protein	85.00
NC_007492	NZ_LT629777	P._asplenii_ATCC_23835.gb		non-ribosomal peptide synthetase	81.98
NC_007492	NZ_CP029608	P._kribbensis_46_2.gb		amino acid adenylation domain-containing protein	83.26
NC_007492	NZ_LT629972	P._fuscovaginae_LMG_2158.gb		non-ribosomal peptide synthetase	82.58
NC_007492	CP027716	P._chlororaphis_PBSt_2.gb		Peptide synthetase	84.55
NC_007492	NZ_CP005969	P._syringae_pv._syringae_B301D.gb		non-ribosomal peptide synthetase	82.68
NC_007492	NZ_LT629777	P._asplenii_ATCC_23835.gb		non-ribosomal peptide synthetase	80.27
NC_007492	NZ_LT629972	P._fuscovaginae_LMG_2158.gb		non-ribosomal peptide synthetase	85.47
NC_007492	NC_004129	P._protegens_Pf_5.gb	ofaB	orfamide A non-ribosomal peptide synthetase OfaB	83.45
NC_007492	NC_012660	P._fluorescens_SBW25.gb		non-ribosomal peptide synthetase	85.50
NC_007492	NZ_LT629972	P._fuscovaginae_LMG_2158.gb		non-ribosomal peptide synthase/polyketide synthase	80.30
NC_007492	NZ_CP009365	P._soli_SJ10.gb		amino acid adenylation domain-containing protein	81.65
NC_007492	NZ_CP006256	P._syringae_pv._syringae_HS191.gb		non-ribosomal peptide synthetase	81.58
NC_007492	NZ_CP077074	P._sessilinigenes_CMR12a.gb		amino acid adenylation domain-containing protein	82.44
NC_007492	NZ_CP077074	P._sessilinigenes_CMR12a.gb		amino acid adenylation domain-containing protein	86.07
NC_007492	NC_007005	P._syringae_pv._syringae_B728a.gb		non-ribosomal peptide synthetase	86.79
NC_007492	NZ_CP007039	P._cichorii_JBC1.gb		non-ribosomal peptide synthetase	84.13
NC_007492	NZ_CP005969	P._syringae_pv._syringae_B301D.gb		non-ribosomal peptide synthase/polyketide synthase	82.24
NC_007492	CP024085	P._putida_E41.gb		non-ribosomal peptide synthetase	82.39
NC_007492	NC_020209	P._poae_RE1_1_14.gb		non-ribosomal peptide synthetase	80.43
NC_007492	CP028826	P._fluorescens_MS82.gb		non-ribosomal peptide synthetase	82.28
NC_007492	NC_020209	P._poae_RE1_1_14.gb		non-ribosomal peptide synthetase	83.66
NC_007492	NZ_JAHSTY010000001	P._azadiae_SWRI103.gb		amino acid adenylation domain-containing protein	83.80
NC_007492	NZ_CP007039	P._cichorii_JBC1.gb		amino acid adenylation domain-containing protein	85.05
NC_007492	CP028826	P._fluorescens_MS82.gb		non-ribosomal peptide synthetase	81.55
NC_007492	NZ_CP020369	P._tolaasii_2192T.gb		amino acid adenylation domain-containing protein	83.04

NC_007492	AE016853	P._syringae_pv._tomato_str._DC3000.gb	syfB	non-ribosomal peptide synthetase SyfB	85.44
NC_007492	CT573326	P._entomophilia_L48.gb		putative non-ribosomal peptide synthetase	86.21
NC_007492	NZ_CP006256	P._syringae_pv._syringae_HS191.gb		non-ribosomal peptide synthetase	80.92
NC_007492	NC_007005	P._syringae_pv._syringae_B728a.gb		non-ribosomal peptide synthetase	82.68
NC_007492	LT629761	P._chlororaphis_DSM_21509.gb		arthrofactin-type cyclic lipopeptide synthetase C	81.75
NC_007492	NZ_LT629972	P._fuscovaginae_LMG_2158.gb		non-ribosomal peptide synthetase	85.96
NC_007492	NZ_CP077074	P._sessilinigenes_CMR12a.gb		amino acid adenylation domain-containing protein	80.61
NC_007492	NZ_LT629777	P._asplenii_ATCC_23835.gb		non-ribosomal peptide synthetase	81.94
NC_007492	NZ_CP020369	P._tolaasii_2192T.gb		amino acid adenylation domain-containing protein	85.15
NC_007492	NZ_LT629972	P._fuscovaginae_LMG_2158.gb		non-ribosomal peptide synthetase	82.96
NC_007492	NZ_LT629778	P._granadensis_LMG_27940.gb		non-ribosomal peptide synthetase	88.51
NC_007492	NZ_AP014522	P._protegens_Cab57.gb	ofaC	orfamide A non-ribosomal peptide synthetase OfaC	86.00
NC_007492	CP028826	P._fluorescens_MS82.gb		non-ribosomal peptide synthetase	93.46
NC_007492	NZ_CP029608	P._kribbensis_46_2.gb		amino acid adenylation domain-containing protein	86.08
NC_007492	CP027716	P._chlororaphis_PBSt_2.gb		Peptide synthetase	84.41
NC_007492	NZ_LT629777	P._asplenii_ATCC_23835.gb		non-ribosomal peptide synthetase	82.78
NC_007492	NZ_CP020369	P._tolaasii_2192T.gb		amino acid adenylation domain-containing protein	84.30
NC_007492	NZ_JAHSTY010000001	P._azadiae_SWRI103.gb		amino acid adenylation domain-containing protein	86.29
NC_007492	NZ_AP014522	P._protegens_Cab57.gb	ofaB	orfamide A non-ribosomal peptide synthetase OfaB	82.83
NC_007492	NZ_CP035088	P._viciae_11K1.gb		non-ribosomal peptide synthetase	82.48
NC_007492	LT629761	P._chlororaphis_DSM_21509.gb		non-ribosomal peptide synthase domain	85.92
NC_007492	NZ_CP005969	P._syringae_pv._syringae_B301D.gb	sypA	syringopeptin non-ribosomal peptide synthetase SypA	85.58
NC_007492	NZ_CP009365	P._soli_SJ10.gb		non-ribosomal peptide synthetase	83.47
NC_007492	NZ_CP005969	P._syringae_pv._syringae_B301D.gb		non-ribosomal peptide synthetase	86.67
NC_007492	LT629761	P._chlororaphis_DSM_21509.gb		arthrofactin-type cyclic lipopeptide synthetase A	80.62
NC_007492	NZ_AP014522	P._protegens_Cab57.gb	ofaA	orfamide A non-ribosomal peptide synthetase OfaA	86.14
NC_007492	LT629761	P._chlororaphis_DSM_21509.gb		arthrofactin-type cyclic lipopeptide synthetase C	85.71
NC_007492	NZ_LT629777	P._asplenii_ATCC_23835.gb		non-ribosomal peptide synthetase	80.46
NC_007492	NZ_CP077074	P._sessilinigenes_CMR12a.gb		amino acid adenylation domain-containing protein	83.50
NC_007492	CT573326	P._entomophilia_L48.gb		putative non-ribosomal peptide synthetase	80.17
NC_007492	NZ_CP011789	P._putida_PC2.gb		non-ribosomal peptide synthetase	84.55
NC_007492	NZ_LT629777	P._asplenii_ATCC_23835.gb		non-ribosomal peptide synthase/polyketide synthase	83.47
NC_007492	NZ_CP007410	P._brassicacearum_DF41.gb		non-ribosomal peptide synthetase	83.85
NC_007492	NC_020209	P._poae_RE1_1_14.gb		non-ribosomal peptide synthetase	84.76
NC_007492	CT573326	P._entomophilia_L48.gb		putative non ribosomal peptide synthetase	82.52
NC_007492	NZ_CP005969	P._syringae_pv._syringae_B301D.gb	sypB	syringopeptin non-ribosomal peptide synthetase SypB	87.13
NC_007492	NC_007005	P._syringae_pv._syringae_B728a.gb	sypC	syringopeptin non-ribosomal peptide synthetase SypC	80.95
NC_007492	NC_007005	P._syringae_pv._syringae_B728a.gb		non-ribosomal peptide synthetase	81.31
NC_007492	NZ_CP035088	P._viciae_11K1.gb		non-ribosomal peptide synthase/polyketide synthase	80.10
NC_007492	NZ_CP005969	P._syringae_pv._syringae_B301D.gb		non-ribosomal peptide synthetase	83.19
NC_007492	NZ_CP006256	P._syringae_pv._syringae_HS191.gb	sypA	syringopeptin non-ribosomal peptide synthetase SypA	84.62

NC_007492	NZ_CP007410	P._brassicacearum_DF41.gb		non-ribosomal peptide synthetase	86.14
NC_007492	NZ_CM001513	P._lactis_SS101.gb		non-ribosomal peptide synthetase	82.52
NC_007492	NZ_CP006256	P._syringae_pv._syringae_HS191.gb		non-ribosomal peptide synthase/polyketide synthase	84.62
NC_007492	NZ_CP020369	P._tolaasii_2192T.gb		non-ribosomal peptide synthetase	80.20
NC_007492	LT629761	P._chlororaphis_DSM_21509.gb		arthrofactin-type cyclic lipopeptide synthetase A	80.62
NC_007492	NZ_LT629972	P._fuscovaginae_LMG_2158.gb		non-ribosomal peptide synthetase	85.09
NC_007492	AE016853	P._syringae_pv._tomato_str._DC3000.gb	syfA	non-ribosomal peptide synthetase SyfA	80.69
NC_007492	NZ_LT629972	P._fuscovaginae_LMG_2158.gb		non-ribosomal peptide synthetase	82.14
NC_007492	NC_007005	P._syringae_pv._syringae_B728a.gb	sypB	syringopeptin non-ribosomal peptide synthetase SypB	82.76
NC_007492	NZ_CP020369	P._tolaasii_2192T.gb		amino acid adenylation domain-containing protein	85.50
NC_007492	NZ_CP020369	P._tolaasii_2192T.gb		phosphopantetheine-binding protein	84.00
NC_007492	NZ_CP005969	P._syringae_pv._syringae_B301D.gb	sypA	syringopeptin non-ribosomal peptide synthetase SypA	85.58
NC_007492	NZ_CP023299	P._mosselii_BS011.gb		amino acid adenylation domain-containing protein	81.56
NC_007492	CP027716	P._chlororaphis_PBSt_2.gb		Siderophore biosynthesis non-ribosomal peptide synthetase	84.21
NC_007492	NC_007005	P._syringae_pv._syringae_B728a.gb	sypA	syringopeptin non-ribosomal peptide synthetase SypA	84.68
NC_007492	NZ_CP005969	P._syringae_pv._syringae_B301D.gb		amino acid adenylation domain-containing protein	83.78
NC_007492	NZ_CP020369	P._tolaasii_2192T.gb		amino acid adenylation domain-containing protein	82.26
NC_007492	NZ_CP007039	P._cichorii_JBC1.gb		non-ribosomal peptide synthase/polyketide synthase	84.86
NC_007492	NZ_CP006256	P._syringae_pv._syringae_HS191.gb		non-ribosomal peptide synthase	82.22
NC_007492	LT629761	P._chlororaphis_DSM_21509.gb		non-ribosomal peptide synthase domain	85.92
NC_007492	NZ_CP029608	P._kribbensis_46_2.gb		non-ribosomal peptide synthetase	88.94
NC_007492	NC_020209	P._poae_RE1_1_14.gb		non-ribosomal peptide synthetase	83.52
NC_007492	NZ_CP007410	P._brassicacearum_DF41.gb		non-ribosomal peptide synthetase	82.67
NC_007492	LT629761	P._chlororaphis_DSM_21509.gb		arthrofactin-type cyclic lipopeptide synthetase B	85.34
NC_007492	NC_004129	P._protegens_Pf_5.gb	ofaC	orfamide A non-ribosomal peptide synthetase OfaC	80.00
NC_007492	NZ_CP011789	P._putida_PC2.gb		non-ribosomal peptide synthetase	82.76
NC_007492	NZ_CP029608	P._kribbensis_46_2.gb		amino acid adenylation domain-containing protein	82.76
NC_007492	CP024085	P._putida_E41.gb		non-ribosomal peptide synthetase	84.82
NC_007492	NZ_CP020369	P._tolaasii_2192T.gb		amino acid adenylation domain-containing protein	80.21
NC_007492	NZ_LT629777	P._asplenii_ATCC_23835.gb		amino acid adenylation domain-containing protein	80.60
NC_007492	NZ_LT629778	P._granadensis_LMG_27940.gb		non-ribosomal peptide synthetase	86.01
NC_007492	NZ_LT629778	P._granadensis_LMG_27940.gb		non-ribosomal peptide synthetase	84.91
NC_007492	NZ_CP005969	P._syringae_pv._syringae_B301D.gb	sypC	syringopeptin non-ribosomal peptide synthetase SypC	81.99
NC_007492	NZ_CP023299	P._mosselii_BS011.gb		non-ribosomal peptide synthetase	87.85
NC_007492	NZ_CP005969	P._syringae_pv._syringae_B301D.gb		non-ribosomal peptide synthase/polyketide synthase	80.20
NC_007492	NZ_CP009365	P._soli_SJ10.gb		non-ribosomal peptide synthetase	82.76
NC_007492	NZ_CP006256	P._syringae_pv._syringae_HS191.gb		amino acid adenylation domain-containing protein	84.68
NC_007492	NZ_LT629972	P._fuscovaginae_LMG_2158.gb		amino acid adenylation domain-containing protein	80.41
NC_007492	NZ_CP009365	P._soli_SJ10.gb		non-ribosomal peptide synthetase	83.19
NC_007492	CP028826	P._fluorescens_MS82.gb		non-ribosomal peptide synthetase	81.43
NC_007492	NC_007005	P._syringae_pv._syringae_B728a.gb		non-ribosomal peptide synthetase	83.19

NC_007492	CT573326	P._entomophilia_L48.gb		putative pyoverdine sidechain peptide synthetase	86.27
NC_007492	NZ_LT629790	P._mediterranea_DSM_16733.gb		non-ribosomal peptide synthetase	80.03
NC_007492	NZ_CP005969	P._syringae_pv._syringae_B301D.gb	sypC	syringopeptin non-ribosomal peptide synthetase SypC	81.99
NC_007492	NZ_LT629778	P._granadensis_LMG_27940.gb		amino acid adenylation domain-containing protein	80.77
NC_007492	NZ_CP029608	P._kribbensis_46_2.gb		amino acid adenylation domain-containing protein	91.23
NC_007492	NZ_CP020369	P._tolaasii_2192T.gb		amino acid adenylation domain-containing protein	80.62
NC_007492	NZ_CP020369	P._tolaasii_2192T.gb		amino acid adenylation domain-containing protein	83.33
NC_007492	NZ_CP007039	P._cichorii_JBC1.gb		non-ribosomal peptide synthetase	84.67
NC_007492	NZ_CP007039	P._cichorii_JBC1.gb		non-ribosomal peptide synthetase	82.63
NC_007492	NZ_CP077074	P._sessilinigenes_CMR12a.gb		amino acid adenylation domain-containing protein	82.68
NC_007492	NZ_CM001513	P._lactis_SS101.gb		non-ribosomal peptide synthetase	86.57
NC_007492	NZ_CP005969	P._syringae_pv._syringae_B301D.gb		amino acid adenylation domain-containing protein	83.78
NC_007492	NZ_CP005969	P._syringae_pv._syringae_B301D.gb		non-ribosomal peptide synthetase	83.19
NC_007492	NZ_LT629777	P._asplenii_ATCC_23835.gb		non-ribosomal peptide synthetase	81.03
NC_007492	NZ_CP009365	P._soli_SJ10.gb		non-ribosomal peptide synthetase	81.34
NC_007492	NZ_CP005969	P._syringae_pv._syringae_B301D.gb	sypB	syringopeptin non-ribosomal peptide synthetase SypB	87.13
NC_007492	AE016853	P._syringae_pv._tomato_str._DC3000.gb		non-ribosomal peptide synthetase, terminal component	82.69
NC_007492	NZ_LT629972	P._fuscovaginae_LMG_2158.gb		non-ribosomal peptide synthetase	81.43
NC_007492	NZ_CP035088	P._viciae_11K1.gb		amino acid adenylation domain-containing protein	85.71
NC_007492	NC_012660	P._fluorescens_SBW25.gb		non-ribosomal peptide synthetase	85.15
NC_007492	NC_021237	P._protegens_CHA0.gb	ofaA	orfamide A non-ribosomal peptide synthetase OfaA	86.14
NC_007492	NZ_CP020369	P._tolaasii_2192T.gb		non-ribosomal peptide synthetase	85.86
NC_007492	NZ_CM001513	P._lactis_SS101.gb		non-ribosomal peptide synthetase	81.63
NC_007492	NZ_CP006256	P._syringae_pv._syringae_HS191.gb		non-ribosomal peptide synthetase	80.39
NC_007492	NZ_CP023299	P._mosselii_BS011.gb		non-ribosomal peptide synthetase	83.14
NC_007492	NC_007005	P._syringae_pv._syringae_B728a.gb		amino acid adenylation domain-containing protein	83.78
NC_007492	CP028826	P._fluorescens_MS82.gb		hypothetical protein	83.74
NC_007492	NZ_CP035088	P._viciae_11K1.gb		amino acid adenylation domain-containing protein	86.11
NC_007492	NZ_LT629790	P._mediterranea_DSM_16733.gb		amino acid adenylation domain-containing protein	84.44
NC_007492	NZ_LT629790	P._mediterranea_DSM_16733.gb		non-ribosomal peptide synthetase	81.09
NC_007492	NZ_CP007410	P._brassicacearum_DF41.gb		non-ribosomal peptide synthetase	80.32
NC_007492	NZ_CP077074	P._sessilinigenes_CMR12a.gb		amino acid adenylation domain-containing protein	80.10
NC_007492	LT629761	P._chlororaphis_DSM_21509.gb		arthrofactin-type cyclic lipopeptide synthetase B	85.34
NC_007492	NZ_CP077074	P._sessilinigenes_CMR12a.gb		non-ribosomal peptide synthetase	83.33
NC_007492	NZ_CP011789	P._putida_PC2.gb		amino acid adenylation domain-containing protein	80.37
NC_007492	NC_021237	P._protegens_CHA0.gb	ofaC	orfamide A non-ribosomal peptide synthetase OfaC	80.36
NC_007492	CP027716	P._chlororaphis_PBSt_2.gb		Non-ribosomal peptide synthetase	84.06
NC_007492	NZ_CP006256	P._syringae_pv._syringae_HS191.gb	sypC	syringopeptin non-ribosomal peptide synthetase SypC	86.14
NC_007492	NC_021237	P._protegens_CHA0.gb	ofaB	orfamide A non-ribosomal peptide synthetase OfaB	83.33
NC_007492	NC_012660	P._fluorescens_SBW25.gb		non-ribosomal peptide synthetase	85.07
NC_007492	CT573326	P._entomophilia_L48.gb		putative non-ribosomal peptide synthetase, terminal component	81.14

NC_007492	NC_004129	P._protegens_Pf_5.gb	ofaA	orfamide A non-ribosomal peptide synthetase OfaA	85.15
NC_007492	NZ_JAHSTY010000001	P._azadiae_SWRI103.gb		amino acid adenylation domain-containing protein	84.40
NC_007492	NC_020209	P._poae_RE1_1_14.gb		non-ribosomal peptide synthetase	81.03
NC_007492	NC_020209	P._poae_RE1_1_14.gb		non-ribosomal peptide synthetase	81.40
NC_007492	NZ_CP020369	P._tolaasii_2192T.gb		amino acid adenylation domain-containing protein	86.00
NC_007492	NZ_CP077074	P._sessilinigenes_CMR12a.gb		amino acid adenylation domain-containing protein	80.90
NC_007492	CP028826	P._fluorescens_MS82.gb		non-ribosomal peptide synthetase	81.00
NC_007492	NZ_CP007039	P._cichorii_JBC1.gb		amino acid adenylation domain-containing protein	83.94
NC_007492	NZ_LT629972	P._fuscovaginae_LMG_2158.gb		non-ribosomal peptide synthetase	85.98
NC_007492	AE016853	P._syringae_pv._tomato_str._DC3000.gb	syfB	non-ribosomal peptide synthetase SyfB	82.05
NC_007492	NC_007005	P._syringae_pv._syringae_B728a.gb		non-ribosomal peptide synthetase	86.67
NC_007492	NZ_LT629972	P._fuscovaginae_LMG_2158.gb		non-ribosomal peptide synthetase	84.96
NC_007492	NC_020209	P._poae_RE1_1_14.gb		non-ribosomal peptide synthetase	81.25
NC_007492	LT629761	P._chlororaphis_DSM_21509.gb		arthrofactin-type cyclic lipopeptide synthetase C	85.71
NC_007492	CT573326	P._entomophilia_L48.gb		putative non-ribosomal peptide synthetase	84.87
NC_007492	NZ_CP077074	P._sessilinigenes_CMR12a.gb		amino acid adenylation domain-containing protein	83.92
NC_007492	NZ_LT629972	P._fuscovaginae_LMG_2158.gb		non-ribosomal peptide synthetase	80.60
NC_007492	NZ_CP020369	P._tolaasii_2192T.gb		amino acid adenylation domain-containing protein	82.05
NC_007492	NZ_CP020369	P._tolaasii_2192T.gb		AMP-binding protein	87.23
NC_007492	NZ_LT629777	P._asplenii_ATCC_23835.gb		non-ribosomal peptide synthetase	81.75
NC_007492	NZ_CP029608	P._kribbensis_46_2.gb		amino acid adenylation domain-containing protein	82.01
NC_007492	CP028826	P._fluorescens_MS82.gb		non-ribosomal peptide synthetase	80.09
NC_007492	NZ_AP014522	P._protegens_Cab57.gb	ofaC	orfamide A non-ribosomal peptide synthetase OfaC	81.09
NC_007492	NZ_LT629778	P._granadensis_LMG_27940.gb		non-ribosomal peptide synthetase	82.91
NC_007492	CP027716	P._chlororaphis_PBSt_2.gb		Peptide synthetase	81.60
NC_007492	NZ_LT629777	P._asplenii_ATCC_23835.gb		non-ribosomal peptide synthetase	85.34
NC_007492	NZ_JAHSTY010000001	P._azadiae_SWRI103.gb		amino acid adenylation domain-containing protein	80.31
NC_007492	CP027716	P._chlororaphis_PBSt_2.gb		Peptide synthetase	80.13
NC_007492	NZ_LT629972	P._fuscovaginae_LMG_2158.gb		non-ribosomal peptide synthetase	82.48
NC_007492	NZ_LT629790	P._mediterranea_DSM_16733.gb		non-ribosomal peptide synthetase	80.10
NC_007492	NZ_LT629972	P._fuscovaginae_LMG_2158.gb		non-ribosomal peptide synthetase	80.46
NC_007492	NC_004129	P._protegens_Pf_5.gb	ofaB	orfamide A non-ribosomal peptide synthetase OfaB	83.33
NC_007492	NZ_LT629777	P._asplenii_ATCC_23835.gb		non-ribosomal peptide synthetase	84.21
NC_007492	NZ_CP005969	P._syringae_pv._syringae_B301D.gb		non-ribosomal peptide synthetase	86.67
NC_007492	NZ_LT629972	P._fuscovaginae_LMG_2158.gb		non-ribosomal peptide synthetase	82.40
NC_007492	NZ_CP009365	P._soli_SJ10.gb		amino acid adenylation domain-containing protein	85.44
NC_007492	NZ_LT629972	P._fuscovaginae_LMG_2158.gb		non-ribosomal peptide synthase/polyketide synthase	88.60
NC_007492	NC_012660	P._fluorescens_SBW25.gb		non-ribosomal peptide synthetase	84.43
NC_007492	NZ_CP077074	P._sessilinigenes_CMR12a.gb		amino acid adenylation domain-containing protein	83.65
NC_007492	NZ_CP007039	P._cichorii_JBC1.gb		non-ribosomal peptide synthetase	86.06
NC_007492	NZ_CP077074	P._sessilinigenes_CMR12a.gb		amino acid adenylation domain-containing protein	83.95

NC_007492	CP024085	P._putida_E41.gb		non-ribosomal peptide synthetase	83.62
NC_007492	NZ_CP005969	P._syringae_pv._syringae_B301D.gb		non-ribosomal peptide synthase/polyketide synthase	80.20
NC_007492	NZ_LT629777	P._asplenii_ATCC_23835.gb		non-ribosomal peptide synthetase	82.58
NC_007492	NZ_CP077074	P._sessilinigenes_CMR12a.gb		amino acid adenylation domain-containing protein	88.04
NC_007492	NZ_CP007039	P._cichorii_JBC1.gb		non-ribosomal peptide synthetase	84.38
NC_007492	NZ_CP077074	P._sessilinigenes_CMR12a.gb		amino acid adenylation domain-containing protein	85.46
NC_007492	NZ_CP005969	P._syringae_pv._syringae_B301D.gb		non-ribosomal peptide synthase/polyketide synthase	80.30
NC_007492	CP024085	P._putida_E41.gb		non-ribosomal peptide synthetase	81.15
NC_007492	NZ_CP009365	P._soli_SJ10.gb		amino acid adenylation domain-containing protein	80.30
NC_007492	NC_012660	P._fluorescens_SBW25.gb		non-ribosomal peptide synthetase	82.62
NC_007492	NZ_LT629972	P._fuscovaginae_LMG_2158.gb		non-ribosomal peptide synthase/polyketide synthase	80.49
NC_007492	NZ_CP006256	P._syringae_pv._syringae_HS191.gb		non-ribosomal peptide synthetase	80.36
NC_007492	NZ_CM001513	P._lactis_SS101.gb		non-ribosomal peptide synthetase	85.71
NC_007492	NZ_LT629777	P._asplenii_ATCC_23835.gb		non-ribosomal peptide synthetase	85.09
NC_007492	NC_004129	P._protegens_Pf_5.gb	ofaB	orfamide A non-ribosomal peptide synthetase OfaB	81.76
NC_007492	NZ_LT629972	P._fuscovaginae_LMG_2158.gb		non-ribosomal peptide synthetase	80.46
NC_007492	NZ_CP005969	P._syringae_pv._syringae_B301D.gb		non-ribosomal peptide synthetase	85.09
NC_007492	NZ_JAHSTY010000001	P._azadiae_SWRI103.gb		amino acid adenylation domain-containing protein	85.15
NC_007492	NZ_LT629777	P._asplenii_ATCC_23835.gb		non-ribosomal peptide synthetase	83.49
NC_007492	NZ_LT629790	P._mediterranea_DSM_16733.gb		non-ribosomal peptide synthetase	87.13
NC_007492	CP027716	P._chlororaphis_PBSt_2.gb		Peptide synthetase	86.07
NC_007492	NZ_AP014522	P._protegens_Cab57.gb	ofaC	orfamide A non-ribosomal peptide synthetase OfaC	83.19
NC_007492	CP028826	P._fluorescens_MS82.gb		non-ribosomal peptide synthetase	80.97
NC_007492	NZ_LT629778	P._granadensis_LMG_27940.gb		non-ribosomal peptide synthetase	82.35
NC_007492	CP027716	P._chlororaphis_PBSt_2.gb		Peptide synthetase	84.40
NC_007492	NZ_CP077074	P._sessilinigenes_CMR12a.gb		amino acid adenylation domain-containing protein	83.33
NC_007492	NZ_CP020369	P._tolaasii_2192T.gb		amino acid adenylation domain-containing protein	80.46
NC_007492	NZ_LT629972	P._fuscovaginae_LMG_2158.gb		non-ribosomal peptide synthetase	83.42
NC_007492	NZ_CP020369	P._tolaasii_2192T.gb		AMP-binding protein	87.08
NC_007492	AE016853	P._syringae_pv._tomato_str._DC3000.gb	syfB	non-ribosomal peptide synthetase SyfB	80.77
NC_007492	NC_007005	P._syringae_pv._syringae_B728a.gb		non-ribosomal peptide synthetase	80.74
NC_007492	NZ_LT629972	P._fuscovaginae_LMG_2158.gb		non-ribosomal peptide synthetase	84.96
NC_007492	LT629761	P._chlororaphis_DSM_21509.gb		arthrofactin-type cyclic lipopeptide synthetase C	81.88
NC_007492	NZ_JAHSTY010000001	P._azadiae_SWRI103.gb		amino acid adenylation domain-containing protein	81.59
NC_007492	NC_020209	P._poae_RE1_1_14.gb		non-ribosomal peptide synthetase	84.09
NC_007492	NC_020209	P._poae_RE1_1_14.gb		non-ribosomal peptide synthetase	82.15
NC_007492	NZ_CP077074	P._sessilinigenes_CMR12a.gb		amino acid adenylation domain-containing protein	82.98
NC_007492	NZ_CP020369	P._tolaasii_2192T.gb		amino acid adenylation domain-containing protein	87.08
NC_007492	NZ_LT629972	P._fuscovaginae_LMG_2158.gb		non-ribosomal peptide synthetase	85.98
NC_007492	NZ_CP007039	P._cichorii_JBC1.gb		amino acid adenylation domain-containing protein	82.63
NC_007492	CP028826	P._fluorescens_MS82.gb		non-ribosomal peptide synthetase	85.58

NC_007492	CT573326	P._entomophilia_L48.gb		putative non-ribosomal peptide synthetase, terminal component	82.98
NC_007492	NC_004129	P._protegens_Pf_5.gb	ofaA	orfamide A non-ribosomal peptide synthetase OfaA	84.58
NC_007492	NZ_CP077074	P._sessilinigenes_CMR12a.gb		non-ribosomal peptide synthetase	83.33
NC_007492	NC_021237	P._protegens_CHA0.gb	ofaC	orfamide A non-ribosomal peptide synthetase OfaC	80.00
NC_007492	NZ_CP011789	P._putida_PC2.gb		amino acid adenylation domain-containing protein	82.43
NC_007492	NZ_CP006256	P._syringae_pv._syringae_HS191.gb	sypC	syringopeptin non-ribosomal peptide synthetase SypC	86.34
NC_007492	CP027716	P._chlororaphis_PBSt_2.gb		Non-ribosomal peptide synthetase	84.06
NC_007492	NC_012660	P._fluorescens_SBW25.gb		non-ribosomal peptide synthetase	83.25
NC_007492	NC_021237	P._protegens_CHA0.gb	ofaB	orfamide A non-ribosomal peptide synthetase OfaB	81.56
NC_007492	LT629761	P._chlororaphis_DSM_21509.gb		arthrofactin-type cyclic lipopeptide synthetase B	86.15
NC_007492	NZ_LT629790	P._mediterranea_DSM_16733.gb		amino acid adenylation domain-containing protein	86.12
NC_007492	NZ_CP007410	P._brassicacearum_DF41.gb		non-ribosomal peptide synthetase	80.63
NC_007492	NZ_LT629790	P._mediterranea_DSM_16733.gb		non-ribosomal peptide synthetase	85.05
NC_007492	NZ_CP077074	P._sessilinigenes_CMR12a.gb		amino acid adenylation domain-containing protein	81.46
NC_007492	NZ_CM001513	P._lactis_SS101.gb		non-ribosomal peptide synthetase	81.95
NC_007492	NZ_CP020369	P._tolaasii_2192T.gb		non-ribosomal peptide synthetase	82.10
NC_007492	NZ_CP023299	P._mosselii_BS011.gb		non-ribosomal peptide synthetase	86.98
NC_007492	NC_007005	P._syringae_pv._syringae_B728a.gb		amino acid adenylation domain-containing protein	83.81
NC_007492	NZ_CP006256	P._syringae_pv._syringae_HS191.gb		non-ribosomal peptide synthetase	83.21
NC_007492	CP028826	P._fluorescens_MS82.gb		hypothetical protein	92.14
NC_007492	NC_021237	P._protegens_CHA0.gb	ofaA	orfamide A non-ribosomal peptide synthetase OfaA	84.11
NC_007492	NZ_CP005969	P._syringae_pv._syringae_B301D.gb	sypB	syringopeptin non-ribosomal peptide synthetase SypB	83.72
NC_007492	NZ_CP009365	P._soli_SJ10.gb		non-ribosomal peptide synthetase	81.25
NC_007492	CP024085	P._putida_E41.gb		non-ribosomal peptide synthetase	84.74
NC_007492	NZ_LT629972	P._fuscovaginae_LMG_2158.gb		non-ribosomal peptide synthetase	84.48
NC_007492	NZ_CP035088	P._viciae_11K1.gb		amino acid adenylation domain-containing protein	83.60
NC_007492	AE016853	P._syringae_pv._tomato_str._DC3000.gb		non-ribosomal peptide synthetase, terminal component	82.69
NC_007492	NC_012660	P._fluorescens_SBW25.gb		non-ribosomal peptide synthetase	82.46
NC_007492	NZ_CP005969	P._syringae_pv._syringae_B301D.gb		amino acid adenylation domain-containing protein	83.81
NC_007492	NZ_LT629777	P._asplenii_ATCC_23835.gb		non-ribosomal peptide synthetase	82.90
NC_007492	NZ_CP005969	P._syringae_pv._syringae_B301D.gb		non-ribosomal peptide synthetase	80.71
NC_007492	NZ_CP007039	P._cichorii_JBC1.gb		non-ribosomal peptide synthetase	85.34
NC_007492	NZ_CP007039	P._cichorii_JBC1.gb		non-ribosomal peptide synthetase	84.38
NC_007492	NZ_CM001513	P._lactis_SS101.gb		non-ribosomal peptide synthetase	83.77
NC_007492	NZ_LT629778	P._granadensis_LMG_27940.gb		amino acid adenylation domain-containing protein	83.26
NC_007492	NZ_CP005969	P._syringae_pv._syringae_B301D.gb	sypC	syringopeptin non-ribosomal peptide synthetase SypC	84.54
NC_007492	NZ_LT629790	P._mediterranea_DSM_16733.gb		non-ribosomal peptide synthetase	81.99
NC_007492	NZ_CP029608	P._kribbensis_46_2.gb		amino acid adenylation domain-containing protein	87.95
NC_007492	NZ_CP020369	P._tolaasii_2192T.gb		amino acid adenylation domain-containing protein	85.64
NC_007492	NZ_CP020369	P._tolaasii_2192T.gb		amino acid adenylation domain-containing protein	83.67
NC_007492	NC_007005	P._syringae_pv._syringae_B728a.gb		non-ribosomal peptide synthetase	80.71

NC_007492	CT573326	P._entomophilia_L48.gb		putative pyoverdine sidechain peptide synthetase	86.27
NC_007492	NZ_LT629972	P._fuscovaginae_LMG_2158.gb		amino acid adenylation domain-containing protein	83.13
NC_007492	NZ_LT629790	P._mediterranea_DSM_16733.gb		non-ribosomal peptide synthetase	84.16
NC_007492	NZ_CP005969	P._syringae_pv._syringae_B301D.gb		non-ribosomal peptide synthase/polyketide synthase	80.30
NC_007492	NZ_CP009365	P._soli_SJ10.gb		non-ribosomal peptide synthetase	85.95
NC_007492	NZ_CP006256	P._syringae_pv._syringae_HS191.gb		amino acid adenylation domain-containing protein	82.38
NC_007492	NZ_LT629778	P._granadensis_LMG_27940.gb		non-ribosomal peptide synthetase	83.76
NC_007492	NZ_LT629777	P._asplenii_ATCC_23835.gb		amino acid adenylation domain-containing protein	82.18
NC_007492	NZ_LT629778	P._granadensis_LMG_27940.gb		non-ribosomal peptide synthetase	84.91
NC_007492	NZ_CP005969	P._syringae_pv._syringae_B301D.gb	sypC	syringopeptin non-ribosomal peptide synthetase SypC	84.54
NC_007492	NZ_CP023299	P._mosselii_BS011.gb		non-ribosomal peptide synthetase	84.96
NC_007492	NZ_CP029608	P._kribbensis_46_2.gb		amino acid adenylation domain-containing protein	85.15
NC_007492	NZ_CP011789	P._putida_PC2.gb		non-ribosomal peptide synthetase	81.31
NC_007492	CP024085	P._putida_E41.gb		non-ribosomal peptide synthetase	84.82
NC_007492	NZ_CP020369	P._tolaasii_2192T.gb		amino acid adenylation domain-containing protein	80.55
NC_007492	NZ_CP007410	P._brassicacearum_DF41.gb		amino acid adenylation domain-containing protein	80.73
NC_007492	LT629761	P._chlororaphis_DSM_21509.gb		non-ribosomal peptide synthase domain	85.92
NC_007492	NZ_CP006256	P._syringae_pv._syringae_HS191.gb		non-ribosomal peptide synthase	80.71
NC_007492	NZ_CP007039	P._cichorii_JBC1.gb		non-ribosomal peptide synthase/polyketide synthase	83.59
NC_007492	NZ_CP029608	P._kribbensis_46_2.gb		non-ribosomal peptide synthetase	90.17
NC_007492	LT629761	P._chlororaphis_DSM_21509.gb		arthrofactin-type cyclic lipopeptide synthetase B	86.15
NC_007492	NC_020209	P._poae_RE1_1_14.gb		non-ribosomal peptide synthetase	82.18
NC_007492	NZ_CP007410	P._brassicacearum_DF41.gb		non-ribosomal peptide synthetase	86.96
NC_007492	NC_004129	P._protegens_Pf_5.gb	ofaC	orfamide A non-ribosomal peptide synthetase OfaC	83.67
NC_007492	NZ_CP005969	P._syringae_pv._syringae_B301D.gb	sypA	syringopeptin non-ribosomal peptide synthetase SypA	85.05
NC_007492	CP027716	P._chlororaphis_PBSt_2.gb		Siderophore biosynthesis non-ribosomal peptide synthetase	81.29
NC_007492	NZ_CP023299	P._mosselii_BS011.gb		amino acid adenylation domain-containing protein	85.09
NC_007492	NC_007005	P._syringae_pv._syringae_B728a.gb	sypA	syringopeptin non-ribosomal peptide synthetase SypA	87.50
NC_007492	NZ_CP005969	P._syringae_pv._syringae_B301D.gb		amino acid adenylation domain-containing protein	83.81
NC_007492	NC_007005	P._syringae_pv._syringae_B728a.gb	sypB	syringopeptin non-ribosomal peptide synthetase SypB	87.79
NC_007492	NZ_CP020369	P._tolaasii_2192T.gb		amino acid adenylation domain-containing protein	81.43
NC_007492	NZ_CP006256	P._syringae_pv._syringae_HS191.gb	sypA	syringopeptin non-ribosomal peptide synthetase SypA	84.86
NC_007492	NZ_CP007410	P._brassicacearum_DF41.gb		non-ribosomal peptide synthetase	80.99
NC_007492	NZ_CP006256	P._syringae_pv._syringae_HS191.gb		non-ribosomal peptide synthase/polyketide synthase	83.97
NC_007492	LT629761	P._chlororaphis_DSM_21509.gb		arthrofactin-type cyclic lipopeptide synthetase A	82.91
NC_007492	NZ_CP020369	P._tolaasii_2192T.gb		non-ribosomal peptide synthetase	80.71
NC_007492	AE016853	P._syringae_pv._tomato_str._DC3000.gb	syfA	non-ribosomal peptide synthetase SyfA	81.43
NC_007492	NZ_LT629972	P._fuscovaginae_LMG_2158.gb		non-ribosomal peptide synthetase	84.91
NC_007492	NZ_CP035088	P._viciae_11K1.gb		non-ribosomal peptide synthase/polyketide synthase	86.00
NC_007492	NZ_CP005969	P._syringae_pv._syringae_B301D.gb		non-ribosomal peptide synthetase	80.71
NC_007492	CT573326	P._entomophilia_L48.gb		putative non ribosomal peptide synthetase	84.04

NC_007492	NC_020209	P._poae_RE1_1_14.gb		non-ribosomal peptide synthetase	84.16
NC_007492	NZ_CP005969	P._syringae_pv._syringae_B301D.gb	sypB	syringopeptin non-ribosomal peptide synthetase SypB	83.72
NC_007492	NC_007005	P._syringae_pv._syringae_B728a.gb	sypC	syringopeptin non-ribosomal peptide synthetase SypC	84.54
NC_007492	NC_007005	P._syringae_pv._syringae_B728a.gb		non-ribosomal peptide synthetase	81.41
NC_007492	NZ_LT629777	P._asplenii_ATCC_23835.gb		non-ribosomal peptide synthetase	80.46
NC_007492	CT573326	P._entomophilia_L48.gb		putative non-ribosomal peptide synthetase	83.49
NC_007492	NZ_CP077074	P._sessilinigenes_CMR12a.gb		amino acid adenylation domain-containing protein	84.58
NC_007492	NZ_LT629777	P._asplenii_ATCC_23835.gb		non-ribosomal peptide synthase/polyketide synthase	81.10
NC_007492	NZ_CP011789	P._putida_PC2.gb		non-ribosomal peptide synthetase	83.65
NC_007492	NZ_CP007410	P._brassicacearum_DF41.gb		non-ribosomal peptide synthetase	83.50
NC_007492	NZ_AP014522	P._protegens_Cab57.gb	ofaB	orfamide A non-ribosomal peptide synthetase OfaB	80.96
NC_007492	NZ_JAHSTY010000001	P._azadiae_SWRI103.gb		amino acid adenylation domain-containing protein	83.37
NC_007492	NZ_CP035088	P._viciae_11K1.gb		non-ribosomal peptide synthetase	86.00
NC_007492	LT629761	P._chlororaphis_DSM_21509.gb		arthrofactin-type cyclic lipopeptide synthetase A	82.91
NC_007492	NZ_CP005969	P._syringae_pv._syringae_B301D.gb		non-ribosomal peptide synthetase	85.09
NC_007492	NZ_CP005969	P._syringae_pv._syringae_B301D.gb	sypA	syringopeptin non-ribosomal peptide synthetase SypA	85.05
NC_007492	LT629761	P._chlororaphis_DSM_21509.gb		non-ribosomal peptide synthase domain	85.92
NC_007492	LT629761	P._chlororaphis_DSM_21509.gb		arthrofactin-type cyclic lipopeptide synthetase C	81.88
NC_007492	NZ_AP014522	P._protegens_Cab57.gb	ofaA	orfamide A non-ribosomal peptide synthetase OfaA	84.11
NC_007492	NZ_LT629777	P._asplenii_ATCC_23835.gb		non-ribosomal peptide synthetase	82.78
NC_007492	NZ_CP020369	P._tolaasii_2192T.gb		amino acid adenylation domain-containing protein	84.30
NC_007492	NZ_CP077074	P._sessilinigenes_CMR12a.gb	macA	macrolide transporter subunit MacA	82.13
NC_007492	NC_007005	P._syringae_pv._syringae_B728a.gb	macA	macrolide transporter subunit MacA	82.20
NC_007492	NZ_JAHSTY010000001	P._azadiae_SWRI103.gb	macA	macrolide transporter subunit MacA	84.83
NC_007492	NC_020209	P._poae_RE1_1_14.gb		macrolide transporter subunit MacA	82.05
NC_007492	NZ_CP007039	P._cichorii_JBC1.gb	macA	macrolide transporter subunit MacA	84.62
NC_007492	NZ_CP007039	P._cichorii_JBC1.gb	macA	macrolide transporter subunit MacA	82.08
NC_007492	LT629761	P._chlororaphis_DSM_21509.gb		membrane fusion protein, macrolide-specific efflux system	84.89
NC_007492	NC_021237	P._protegens_CHA0.gb	macA	macrolide transporter subunit MacA	84.03
NC_007492	CT573326	P._entomophilia_L48.gb	macA	macrolide efflux protein MacA	83.81
NC_007492	LT629761	P._chlororaphis_DSM_21509.gb		membrane fusion protein, macrolide-specific efflux system	84.89
NC_007492	AE016853	P._syringae_pv._tomato_str._DC3000.gb	syfC	syringafactin efflux protein SyfC	84.42
NC_007492	NZ_LT629790	P._mediterranea_DSM_16733.gb	macA	macrolide transporter subunit MacA	82.62
NC_007492	NZ_CP006256	P._syringae_pv._syringae_HS191.gb	macA	macrolide transporter subunit MacA	85.90
NC_007492	NZ_CP007410	P._brassicacearum_DF41.gb	macA	macrolide transporter subunit MacA	83.59
NC_007492	CP027716	P._chlororaphis_PBSt_2.gb		Macrolide-specific efflux protein MacA	84.62
NC_007492	NZ_CP029608	P._kribbensis_46_2.gb		macrolide transporter subunit MacA	93.65
NC_007492	NZ_CP009365	P._soli_SJ10.gb	macA	macrolide transporter subunit MacA	80.30
NC_007492	NC_004129	P._protegens_Pf_5.gb	macA	macrolide transporter subunit MacA	83.94
NC_007492	NZ_CM001513	P._lactis_SS101.gb		macrolide transporter subunit MacA	83.54
NC_007492	NZ_CP020369	P._tolaasii_2192T.gb	macA	macrolide transporter subunit MacA	83.11

NC_007492	NZ_LT629778	P._granadensis_LMG_27940.gb	macA	macrolide transporter subunit MacA	85.02
NC_007492	CP024085	P._putida_E41.gb		macrolide transporter subunit MacA	80.90
NC_007492	NZ_LT629777	P._asplenii_ATCC_23835.gb	macA	macrolide transporter subunit MacA	82.58
NC_007492	NZ_AP014522	P._protegens_Cab57.gb	macA	macrolide transporter subunit MacA	84.22
NC_007492	NZ_LT629972	P._fuscovaginae_LMG_2158.gb	macA	macrolide transporter subunit MacA	82.14
NC_007492	NZ_CP023299	P._mosselii_BS011.gb	macA	macrolide transporter subunit MacA	80.21
NC_007492	NC_012660	P._fluorescens_SBW25.gb		macrolide transporter subunit MacA	82.90
NC_007492	CP028826	P._fluorescens_MS82.gb		macrolide transporter subunit MacA	93.73
NC_007492	NZ_CP005969	P._syringae_pv._syringae_B301D.gb	macA	macrolide transporter subunit MacA	82.20
NC_007492	NZ_CP035088	P._viciae_11K1.gb	macA	macrolide transporter subunit MacA	82.69
NC_007492	NZ_CP005969	P._syringae_pv._syringae_B301D.gb	macA	macrolide transporter subunit MacA	82.20
NC_007492	NZ_CP020369	P._tolaasii_2192T.gb	macA	macrolide transporter subunit MacA	85.05
NC_007492	NZ_CP077074	P._sessilinigenes_CMR12a.gb	macA	macrolide transporter subunit MacA	82.54
NC_007492	NC_012660	P._fluorescens_SBW25.gb		MacB family efflux pump subunit	83.39
NC_007492	AE016853	P._syringae_pv._tomato_str._DC3000.gb	syfD	syringafactin efflux protein SyfD	83.65
NC_007492	NZ_CP005969	P._syringae_pv._syringae_B301D.gb		MacB family efflux pump subunit	80.37
NC_007492	NZ_CP006256	P._syringae_pv._syringae_HS191.gb		MacB family efflux pump subunit	82.62
NC_007492	NZ_CP020369	P._tolaasii_2192T.gb		MacB family efflux pump subunit	82.61
NC_007492	NC_020209	P._poae_RE1_1_14.gb		MacB family efflux pump subunit	84.69
NC_007492	CP028826	P._fluorescens_MS82.gb		macrolide ABC transporter permease/ATP-binding protein MacB	93.20
NC_007492	NZ_LT629778	P._granadensis_LMG_27940.gb		MacB family efflux pump subunit	86.55
NC_007492	NC_004129	P._protegens_Pf_5.gb		MacB family efflux pump subunit	84.23
NC_007492	NZ_CP005969	P._syringae_pv._syringae_B301D.gb		MacB family efflux pump subunit	80.37
NC_007492	CT573326	P._entomophilia_L48.gb	macB	macrolide ABC efflux protein MacB	83.07
NC_007492	NZ_CP009365	P._soli_SJ10.gb		MacB family efflux pump subunit	83.15
NC_007492	NZ_LT629777	P._asplenii_ATCC_23835.gb		MacB family efflux pump subunit	82.55
NC_007492	NZ_LT629777	P._asplenii_ATCC_23835.gb		MacB family efflux pump subunit	83.37
NC_007492	LT629761	P._chlororaphis_DSM_21509.gb		macrolide transport system ATP-binding/permease protein	87.19
NC_007492	NZ_CM001513	P._lactis_SS101.gb		MacB family efflux pump subunit	84.89
NC_007492	CP027716	P._chlororaphis_PBSt_2.gb		Macrolide export ATP-binding/permease protein MacB	87.07
NC_007492	NZ_CP007039	P._cichorii_JBC1.gb		MacB family efflux pump subunit	82.62
NC_007492	NZ_CP077074	P._sessilinigenes_CMR12a.gb		MacB family efflux pump subunit	83.23
NC_007492	NZ_CP011789	P._putida_PC2.gb		MacB family efflux pump subunit	82.16
NC_007492	NZ_CP077074	P._sessilinigenes_CMR12a.gb		MacB family efflux pump subunit	84.09
NC_007492	NC_007005	P._syringae_pv._syringae_B728a.gb		MacB family efflux pump subunit	80.51
NC_007492	LT629761	P._chlororaphis_DSM_21509.gb		macrolide transport system ATP-binding/permease protein	87.19
NC_007492	NC_021237	P._protegens_CHA0.gb		MacB family efflux pump subunit	84.41
NC_007492	NZ_CP029608	P._kribbensis_46_2.gb	macB	MacB family efflux pump subunit	92.74
NC_007492	NZ_LT629972	P._fuscovaginae_LMG_2158.gb		MacB family efflux pump subunit	87.04
NC_007492	NZ_CP007410	P._brassicacearum_DF41.gb		MacB family efflux pump subunit	82.72
NC_007492	NZ_CP007039	P._cichorii_JBC1.gb		MacB family efflux pump subunit	81.41

NC_007492	NZ_CP023299	P._mosselii_BS011.gb	MacB family efflux pump subunit	83.57
NC_007492	NZ_AP014522	P._protegens_Cab57.gb	MacB family efflux pump subunit	84.14
NC_007492	NZ_CP020369	P._tolaasii_2192T.gb	MacB family efflux pump subunit	84.81
NC_007492	NZ_LT629972	P._fuscovaginae_LMG_2158.gb	MacB family efflux pump subunit	83.46
NC_007492	CP024085	P._putida_E41.gb	macrolide ABC transporter permease/ATP-binding protein MacB	84.92
NC_007492	NZ_JAHSTY010000001	P._azadiae_SWRI103.gb	MacB family efflux pump subunit	87.02
NC_007492	NZ_CP035088	P._viciae_11K1.gb	MacB family efflux pump subunit	83.25
NC_007492	NZ_CM001513	P._lactis_SS101.gb	LuxR family transcriptional regulator	89.17
NC_007492	NZ_LT629972	P._fuscovaginae_LMG_2158.gb	LuxR C-terminal-related transcriptional regulator	83.73
NC_007492	NZ_CP029608	P._kribbensis_46_2.gb	helix-turn-helix transcriptional regulator	83.46
NC_007492	CP027716	P._chlororaphis_PBSt_2.gb	Transcriptional regulator, LuxR family	87.04
NC_007492	NZ_CP007410	P._brassicacearum_DF41.gb	helix-turn-helix transcriptional regulator	82.99
NC_007492	NC_020209	P._poae_RE1_1_14.gb	LuxR family transcriptional regulator	83.44
NC_007492	NZ_AP014522	P._protegens_Cab57.gb	helix-turn-helix transcriptional regulator	81.07
NC_007492	NC_021237	P._protegens_CHA0.gb	LuxR family transcriptional regulator	81.66
NC_007492	NZ_LT629777	P._asplenii_ATCC_23835.gb	LuxR C-terminal-related transcriptional regulator	83.73
NC_007492	NC_012660	P._fluorescens_SBW25.gb	LuxR family transcriptional regulator	87.50
NC_007492	LT629761	P._chlororaphis_DSM_21509.gb	transcriptional regulator, LuxR family	85.19
NC_007492	NZ_JAHSTY010000001	P._azadiae_SWRI103.gb	helix-turn-helix transcriptional regulator	86.01
NC_007492	LT629761	P._chlororaphis_DSM_21509.gb	transcriptional regulator, LuxR family	85.19
NC_007492	NZ_LT629778	P._granadensis_LMG_27940.gb	helix-turn-helix transcriptional regulator	88.11
NC_007492	NC_004129	P._protegens_Pf_5.gb	helix-turn-helix transcriptional regulator	81.66
NC_007492	NZ_CP020369	P._tolaasii_2192T.gb	helix-turn-helix transcriptional regulator	87.61
NC_007492	NZ_CP011789	P._putida_PC2.gb	LuxR family transcriptional regulator	83.33
NC_007492	NZ_CP077074	P._sessilinigenes_CMR12a.gb	LuxR C-terminal-related transcriptional regulator	85.19
NC_007492	CP028826	P._fluorescens_MS82.gb	LuxR family transcriptional regulator	94.68
NC_007492	NZ_CP029608	P._kribbensis_46_2.gb	amino acid adenylation domain-containing protein	85.48
NC_007492	NC_004129	P._protegens_Pf_5.gb	non-ribosomal peptide synthetase	83.65
NC_007492	NZ_LT629777	P._asplenii_ATCC_23835.gb	non-ribosomal peptide synthetase	81.88
NC_007492	CT573326	P._entomophilia_L48.gb	putative non-ribosomal peptide synthetase, terminal component	81.73
NC_007492	NZ_CP029608	P._kribbensis_46_2.gb	amino acid adenylation domain-containing protein	83.65
NC_007492	NC_007005	P._syringae_pv._syringae_B728a.gb	non-ribosomal peptide synthetase	84.59
NC_007492	NZ_CP005969	P._syringae_pv._syringae_B301D.gb	amino acid adenylation domain-containing protein	84.89
NC_007492	NZ_CP029608	P._kribbensis_46_2.gb	amino acid adenylation domain-containing protein	83.97
NC_007492	NZ_CP023299	P._mosselii_BS011.gb	amino acid adenylation domain-containing protein	81.14
NC_007492	NZ_LT629777	P._asplenii_ATCC_23835.gb	non-ribosomal peptide synthetase	81.29
NC_007492	NZ_AP014522	P._protegens_Cab57.gb	non-ribosomal peptide synthetase	84.00
NC_007492	NC_021237	P._protegens_CHA0.gb	non-ribosomal peptide synthetase	83.65
NC_007492	NZ_CP029608	P._kribbensis_46_2.gb	amino acid adenylation domain-containing protein	80.91
NC_007492	NZ_LT629777	P._asplenii_ATCC_23835.gb	non-ribosomal peptide synthetase	83.65
NC_007492	NZ_CP035088	P._viciae_11K1.gb	non-ribosomal peptide synthase/polyketide synthase	84.68

NC_007492	NC_012660	P._fluorescens_SBW25.gb		non-ribosomal peptide synthetase	84.16
NC_007492	CP027716	P._chlororaphis_PBSt_2.gb		Peptide synthetase	82.32
NC_007492	NZ_LT629972	P._fuscovaginae_LMG_2158.gb		non-ribosomal peptide synthetase	82.91
NC_007492	NZ_CP005969	P._syringae_pv._syringae_B301D.gb		amino acid adenylation domain-containing protein	84.89
NC_007492	CT573326	P._entomophilia_L48.gb		putative non-ribosomal peptide synthetase	82.63
NC_007492	CP028826	P._fluorescens_MS82.gb		non-ribosomal peptide synthetase	80.91
NC_007492	NZ_LT629972	P._fuscovaginae_LMG_2158.gb		non-ribosomal peptide synthetase	83.23
NC_007492	LT629761	P._chlororaphis_DSM_21509.gb		arthrofactin-type cyclic lipopeptide synthetase B	82.32
NC_007492	NZ_CP006256	P._syringae_pv._syringae_HS191.gb		non-ribosomal peptide synthetase	84.89
NC_007492	NZ_LT629777	P._asplenii_ATCC_23835.gb		non-ribosomal peptide synthetase	83.47
NC_007492	NZ_CP005969	P._syringae_pv._syringae_B301D.gb	sypB	syringopeptin non-ribosomal peptide synthetase SypB	83.18
NC_007492	NZ_CP029608	P._kribbensis_46_2.gb		amino acid adenylation domain-containing protein	83.33
NC_007492	NZ_CP007410	P._brassicacearum_DF41.gb		non-ribosomal peptide synthetase	83.49
NC_007492	NZ_LT629972	P._fuscovaginae_LMG_2158.gb		non-ribosomal peptide synthetase	81.73
NC_007492	NZ_LT629777	P._asplenii_ATCC_23835.gb		non-ribosomal peptide synthetase	86.27
NC_007492	NZ_LT629972	P._fuscovaginae_LMG_2158.gb		non-ribosomal peptide synthetase	81.44
NC_007492	CT573326	P._entomophilia_L48.gb		putative non ribosomal peptide synthetase	81.12
NC_007492	CP028826	P._fluorescens_MS82.gb		non-ribosomal peptide synthetase	84.18
NC_007492	NZ_CP029608	P._kribbensis_46_2.gb		amino acid adenylation domain-containing protein	90.85
NC_007492	NZ_CP005969	P._syringae_pv._syringae_B301D.gb	sypB	syringopeptin non-ribosomal peptide synthetase SypB	83.18
NC_007492	CP027716	P._chlororaphis_PBSt_2.gb		Peptide synthetase	81.01
NC_007492	NC_020209	P._poae_RE1_1_14.gb		non-ribosomal peptide synthetase	84.26
NC_007492	NC_020209	P._poae_RE1_1_14.gb		non-ribosomal peptide synthetase	82.84
NC_007492	CP028826	P._fluorescens_MS82.gb		non-ribosomal peptide synthetase	95.33
NC_007492	CT573326	P._entomophilia_L48.gb	psvA	pyoverdine synthetase A	82.11
NC_007492	NZ_CP006256	P._syringae_pv._syringae_HS191.gb		non-ribosomal peptide synthase	81.06
NC_007492	NZ_CP035088	P._viciae_11K1.gb		non-ribosomal peptide synthetase	83.78
NC_007492	AE016853	P._syringae_pv._tomato_str._DC3000.gb	syfB	non-ribosomal peptide synthetase SyfB	80.75
NC_007492	LT629761	P._chlororaphis_DSM_21509.gb		arthrofactin-type cyclic lipopeptide synthetase B	82.32
NC_007492	NZ_CP006256	P._syringae_pv._syringae_HS191.gb		non-ribosomal peptide synthetase	82.07
NC_007492	CT573326	P._entomophilia_L48.gb		putative non-ribosomal peptide synthetase	82.89
NC_007492	NZ_LT629777	P._asplenii_ATCC_23835.gb		non-ribosomal peptide synthetase	83.04
NC_007492	NZ_CP029608	P._kribbensis_46_2.gb		amino acid adenylation domain-containing protein	90.01
NC_007492	NZ_LT629972	P._fuscovaginae_LMG_2158.gb		non-ribosomal peptide synthetase	81.18
NC_007492	NZ_CP005969	P._syringae_pv._syringae_B301D.gb		amino acid adenylation domain-containing protein	82.05
NC_007492	NZ_CP029608	P._kribbensis_46_2.gb		amino acid adenylation domain-containing protein	84.62
NC_007492	NZ_CM001513	P._lactis_SS101.gb		non-ribosomal peptide synthetase	80.77
NC_007492	NC_007005	P._syringae_pv._syringae_B728a.gb		non-ribosomal peptide synthetase	81.85
NC_007492	NZ_LT629972	P._fuscovaginae_LMG_2158.gb		non-ribosomal peptide synthetase	84.40
NC_007492	NZ_CP020369	P._tolaasii_2192T.gb		non-ribosomal peptide synthetase	82.12
NC_007492	CP028826	P._fluorescens_MS82.gb		non-ribosomal peptide synthetase	95.50

NC_007492	NZ_CP006256	P._syringae_pv._syringae_HS191.gb	non-ribosomal peptide synthetase	83.33
NC_007492	NZ_CP006256	P._syringae_pv._syringae_HS191.gb	non-ribosomal peptide synthetase	82.87
NC_007492	CT573326	P._entomophilia_L48.gb	putative non-ribosomal peptide synthetase	82.77
NC_007492	NZ_CP029608	P._kribbensis_46_2.gb	amino acid adenylation domain-containing protein	84.43
NC_007492	NZ_LT629777	P._asplenii_ATCC_23835.gb	non-ribosomal peptide synthetase	83.33
NC_007492	NC_012660	P._fluorescens_SBW25.gb	non-ribosomal peptide synthetase	81.89
NC_007492	NZ_CP005969	P._syringae_pv._syringae_B301D.gb	non-ribosomal peptide synthetase	81.95
NC_007492	NZ_LT629972	P._fuscovaginae_LMG_2158.gb	non-ribosomal peptide synthetase	81.76
NC_007492	NZ_LT629777	P._asplenii_ATCC_23835.gb	non-ribosomal peptide synthetase	80.11
NC_007492	NC_007005	P._syringae_pv._syringae_B728a.gb	non-ribosomal peptide synthetase	82.69
NC_007492	NZ_CP029608	P._kribbensis_46_2.gb	amino acid adenylation domain-containing protein	80.91
NC_007492	NZ_CP005969	P._syringae_pv._syringae_B301D.gb	non-ribosomal peptide synthetase	81.95
NC_007492	NZ_LT629777	P._asplenii_ATCC_23835.gb	non-ribosomal peptide synthetase	81.76
NC_007492	NZ_CP029608	P._kribbensis_46_2.gb	amino acid adenylation domain-containing protein	84.11
NC_007492	NZ_CP005969	P._syringae_pv._syringae_B301D.gb	amino acid adenylation domain-containing protein	82.05
NC_007492	NZ_LT629778	P._granadensis_LMG_27940.gb	non-ribosomal peptide synthetase	81.10
NC_007492	NC_007005	P._syringae_pv._syringae_B728a.gb	amino acid adenylation domain-containing protein	83.52
NC_007492	NZ_CP005969	P._syringae_pv._syringae_B301D.gb	hybrid non-ribosomal peptide synthetase/type I polyketide synthase	83.52
NC_007492	NZ_LT629777	P._asplenii_ATCC_23835.gb	non-ribosomal peptide synthetase	83.96
NC_007492	CT573326	P._entomophilia_L48.gb	putative Polyketide Synthase (terminal component)	85.71
NC_007492	NZ_CP006256	P._syringae_pv._syringae_HS191.gb	SDR family NAD(P)-dependent oxidoreductase	85.48
NC_007492	NZ_LT629972	P._fuscovaginae_LMG_2158.gb	non-ribosomal peptide synthetase	83.96
NC_007492	CP028826	P._fluorescens_MS82.gb	non-ribosomal peptide synthetase	94.90
NC_007492	NZ_CP005969	P._syringae_pv._syringae_B301D.gb	hybrid non-ribosomal peptide synthetase/type I polyketide synthase	83.52
NC_007492	NZ_CP029608	P._kribbensis_46_2.gb	amino acid adenylation domain-containing protein	83.26
